# Certain fermented dairy foods as a source of multibiotics and multimetabolites: a comprehensive review

**DOI:** 10.3389/fnut.2025.1678150

**Published:** 2025-11-06

**Authors:** Duygu Ağagündüz, Yasemin Ertaş Öztürk, Büşra Ayhan, Tuğçe Bulmuş-Tüccar, Çiler Özenir, Nazlıcan Erdoğan Gövez, Yesim Ozogul, Tuba Esatbeyoglu, Fatih Ozogul

**Affiliations:** 1 Department of Nutrition and Dietetics, Faculty of Health Sciences, Gazi University, Ankara, Türkiye; 2 Department of Nutrition and Dietetics, Faculty of Health Sciences, Ondokuz Mayıs University, Samsun, Türkiye; 3 Arla Innovation Centre, Aarhus, Denmark; 4 Department of Nutrition and Dietetics, Faculty of Health Sciences, Kırıkkale University, Kırıkkale, Türkiye; 5 Department of Seafood Processing Technology, Faculty of Fisheries, Cukurova University, Adana, Türkiye; 6 Department of Molecular Food Chemistry and Food Development, Institute of Food and One Health, Gottfried Wilhelm Leibniz University Hannover, Hannover, Germany; 7 Biotechnology Research and Application Center, Cukurova University, Adana, Türkiye

**Keywords:** fermented dairy foods, biotics, metabolites, nutrition, health

## Abstract

Fermentation, a traditional biotechnological food bioprocessing, has been used for centuries. It enables the preservation of perishable foods and designing a novel food product with different taste and rheological properties. Fermented foods are defined as “foods made through desired microbial growth and enzymatic conversions of food components by The International Scientific Association of Probiotics and Prebiotics (ISAPP). Regarding this, the most popular fermented products are fermented dairy products which are commonly produced by lactic acid fermentation such as fermented milk, yogurt, kefir, sour cream, cultured buttermilk and cheeses, and some novel fermented dairy products. Accumulated literature suggests that fermented dairy products are one of the important sources of some nutritional biotics like probiotics, prebiotics, postbiotics and some bioactive metabolites. At the molecular level, the fermented dairy products’ matrices are composed of hundreds of compounds and various metabolites, including organic acids and derivatives, carbohydrates, lipids and lipidomics, milk fat globule membrane (MFGM), proteins, amino acids, bioactive peptides, nucleic acids, vitamins, minerals, and aroma volatiles, etc. which contribute to their technological and aroma properties. A number of preclinical and clinical studies suggest that these biotics and metabolites have promising health effects as well as their technological benefits. These effects of fermented dairy products significantly vary according to plenty of factors such as the milk types and composition, products’ microorganism profiles, matrix, added ingredients, etc. This comprehensive review focuses on the fermented dairy foods as a source of multibiotics and multimetabolites with technological importance and health-promoting effects on human health.

## 1 Introduction

There is a growing demand on fermented foods globally and the size of the global fermented food and beverages market was US$ 575.6 Billion in 2022 and is expected to increase at a compound annual growth rate of 5.6%, reaching around US$ 989.2 Billion by 2032 (1).

On a global scale, fermented foods make up around one-third of the human diet (2). They are substantial group of foods that valued globally due to their distinct flavors and nutritional attributes. The fermentation process alters certain sensory characteristics of foods. The genetic traits of several microorganisms are accountable for the entire synthesis of metabolites in fermented foods. The bacteria secrete or generate enzymes that decompose complicated substances into simpler ones that may promote health (3). An observational data from The National Health and Nutrition Examination Survey NHANES 2001–2018 showed that 100-g intake of microorganism–containing foods was associated with modest health improvements. Lower systolic blood pressure, C-reactive protein (CRP), blood glycemic parameters (glucose and insulin), lipid profile (lower triglyceride, higher HDL-C), anthropometric measurements linked obesity (waist circumference, body mass index) have been shown (4).

Fermented dairy products play a crucial role in the traditional food cultures of several ethnic communities across the world. These products are made from the unpasteurized or pasteurized milk of various animals such as cows, buffaloes, yaks, camels, goats, and sheep using a process called back-slopping or spontaneous fermentation (5). The Mediterranean diet pyramid recommends consuming modest quantities of dairy products, particularly yogurt and cheese, on a daily basis. This aligns with the global dietary guidelines of consuming 2–3 servings of dairy products per day (6). There is growing evidence that fermented dairy products confer various health benefits to consumers, such as reduced risk of breast and colorectal cancer, lowering serum cholesterol, decreased type 2 diabetes, improved weight maintenance, cardiovascular health, bone health, and gastrointestinal health, boosting immune response, and mitigating cognitive impairment (7–9).

Fermented dairy products are often a natural source and potential carrier of multiple biotics such as probiotics, postbiotics, and prebiotics (10). Probiotics are commonly present in a variety of fermented dairy products, particularly yogurt, kefir, koumiss, and cultured beverages (7) which contribute various biological capabilities and provide health-promoting advantages to individuals. Fermented foods contain beneficial microorganisms, primarily lactic acid bacteria (LAB), as well as non-LAB species like *Propionibacterium freudenreichii* and *Bifidobacterium longum*, and a small number of yeast species, principally *Saccharomyces boulardii* (11). Lactobacillus is the predominant and significant genus, with 51 documented species. The species *Lactobacillus helveticus*, *Lactobacillus kefiranofaciens* subsp. *kefirgranum*, *Lactobacillus delbrueckii*, and *Lentilactobacillus kefiri* are commonly found in kefir, koumiss, tarag, buttermilk, dahi, khoormog, and kurut (12).

Postbiotics are another naturally found biotics in fermented dairy products. They are composed of nonliving microbes and/or their parts that have beneficial effects for the host’s health (13). Lactic acid is the primary metabolite generated during LAB fermentation. Moreover, proteolytic, lipolytic, amylolytic, and esterolytic activity of microorganisms such as *LAB, Pseudomonas, Bacillus, Achromobacter*, etc. appear to be the primary factors driving the metabolic alterations in fermented foods (3). The observed health effects can be linked to a wide variety of functional constituents, including bioactive peptides, polysaccharides, fatty acids, organic acids, vitamins, and *γ*-amino butyric acid (GABA) (9).

Fermented milk products contain and serve as a carrier for prebiotics as well. Indigestible dietary components, lactose, and exopolysaccharides (EPS) contribute to the prebiotic content in fermented dairy products (14). Inulin, fructooligosaccharides, xylooligosaccharides, isomaltooligosaccharides, and wheat fiber have been used into various dairy products, including yogurt and fermented milk products (15). They present health effects as substrates for probiotics and generate antimicrobial peptides, metabolites, growth factors, immunological modulators, and neuroactive compounds. Within the other biotics in fermented dairy products, they contribute to the preservation of human immune function, mitigation of cancer risk, alleviation of allergies, establishment in the gut environment, lowering of cholesterol levels, promote the brain health and enhances gastrointestinal microbiota (16). The probiotic bacteria prompt the host to generate immunoglobulin, specifically IgA, which aids in the elimination of harmful germs from the body. Probiotics emit organic acids and antibacterial peptides that effectively eliminate pathogenic organisms in the gut environment. Probiotics, when included in the human diet, have a crucial role in enhancing digestive, respiratory, and immunological processes while reducing the occurrence of infectious disorders (17).

The diversity and complexity of multibiotics, along with the microorganisms utilized in the fermentation process, make it challenging to fully understand the precise composition and molecular structure of fermented dairy products (18). New analytical techniques and current advancements in mass spectrometry allow for comprehensive data collection and a better understanding of the effects of different raw materials, starter cultures, fermentation temperature, and storage on the metabolite profile. Metabolomics, with its high-throughput and comprehensive metabolite coverage characteristics, has been widely used for identifying chemical composition and quantifying metabolites affected by various specified factors (19). Thus, the integration of high throughput separation techniques and screening aids would greatly improve our understanding of how specific LAB species might be utilized in the dairy business, particularly those with desirable attributes. An analysis of metabolites and their concentration fluctuations during fermentation would enhance our understanding of how to enhance the manufacturing process and create potentially useful substances for health (18). Therefore, this review comprehensively held on the fermented dairy foods as a source of multiple multibiotics and multiomics.

## 2 Fermented milk

Fermented milk, delineated by the fourth version of the Codex Alimentarius Standards (CXS 243–2003); “fermented milk is a milk product obtained by fermentation of milk, which milk may have been manufactured from products obtained from milk with or without compositional modification, by the action of suitable microorganisms and resulting in the reduction of pH with or without coagulation” (20). According to this definition yoghurt, alternate culture yoghurt, acidophilus milk, kefir and koumiss are certain fermented milks. Since yoghurt and kefir are examined in the following sections of the review, traditional and artisanal fermented milk products are mentioned in this section.

Although the exact origins of fermented milk are unclear, it is widely recognized product that has been associated with the earliest stages of human existence (20, 21). Many ancient civilizations, such as Egypt, Mesopotamia, and the Indian subcontinent, independently produced fermented dairy products around the same period. Fermented milk likely emerged in Türkiye, namely in the region of the Anatolian plateau (3,000 B. C.). Indeed, in a recent study archaeochemical investigations from Barcın Höyük (northwestern Anatolia) have shown that by around 6,600 BCE, almost 60% of the pottery shards had milk traces on them (22). Egypt possesses visual and physical evidence of cheese production dating back to 3,000 BCE, as shown in tomb paintings and alabaster jars (23). Additionally, Mesopotamian texts from Sumer and Akkad chronicle butter, cheese, and sour milk by the middle of the third millennium BCE and Dahi from India was originated as coagulated sour milk -an eaten food item- by around 6,000–4,000 BCE (24). Milk contains the necessary nutrients and minerals for bacterial development. Moreover, the protective properties of milk and its lipids create a conducive environment for bacteria to thrive in the challenging conditions of the gastrointestinal system. During the pre-scientific era, fermentation, which is a natural and spontaneous process caused by microorganisms present in the milk, was initially perceived as milk deterioration. However, it was later discovered that fermentation actually preserves the milk’s components and prolongs its storage time. In modern times, traditional fermented milk products are produced using a time-honored method known as the “back-sloping” technique (21). With the discovery of the health-promoting effects of fermented milk with both *in vivo* and human randomized trials (25), its consumption has increased. Currently, the global fermented milk market was anticipated to be valued at US$ 54,760.00 million in 2022 and is expected to reach US$ 67,347.89 million by 2029 (20).

Laban Rayeb, Laban/Zabady, Laban Khad, Labneh, Laban Zeer, Labneh Ambaris/Serdeleh, Labneh Darf, Shanklish/Surk, Ayran, Kishk, Kurut, Tuzlu/Salted Yoghurt are reported as fermented milk or concentrated milk products of Eastern Mediterranean countries mainly Türkiye, Syria, Lebanon, Palestine and Egypt. Although they have different process Limosilactobacillus is the dominant bacterial genus and *Saccharomyces cerevisiae* is the dominant yeast. Bacteria described as probiotics such as *Lactiplantibacillus plantarum*, *Levilactobacillus brevis*, and *Lacticaseibacillus casei* have been identified in most of these fermented milk products (26). Zabady, Lben, Kefir, Doogh, Mast, Chal, Shubat, Rayeb including Laban are popular traditional fermented milks from Middle Eastern and Northern African countries. Along with LAB, according to the different milk type that produced the fermented milk microorganism content varied. Indeed, Shubat and Chal traditionally fermented camel milk, have been reported as including *Pseudomonas putida, Kocuria rosea*, and *Staphylococcus simulans,* as well as *Lusitania and Cryptococcus laurentii* with yeast (27). In the Nordic countries of Northern Europe comprise Denmark, Finland, Iceland, Norway, and Sweden, fermented milks are buttermilk (liquid by-product of butter making), cultured buttermilk, cultured milk mainly fermented by mesophilic LAB belonging to the genera Lactococcus and Leuconostoc (28). Dahi is another traditional yogurt like LAB fermented yak, cow and/or buffalo milk product from India (29). In Central Asia various fermented milk products from cow, mare, camel, yak, reindeer consumed traditionally. Ayran, Kefir, Tan, Qymyz, Shubat, Chal, Khoormog are the examples among fermented milks with a wide range biodiversity (30). The traditional drinkable, viscous or dried fermented milk products are not limited with above but reviewed elsewhere in detail from all over the world (31–33).

The milk fermentation process is influenced by the content of the milk, as well as the selection and amount of starter culture used, along with the addition of probiotics. The fermentation characteristics of various bacterial strains utilized in milk fermentation, such as viable cell count, pH, and titratable acidity, significantly differ. These differences have an impact on the texture, aroma, and sensory aspects of the final fermented products (34). Moreover, the duration of fermentation alters the metabolite and probiotic composition of the milk. The metabolome of probiotic fermented milk, using *Lacticaseibacillus paracasei* PC-01 and *Bifidobacterium adolescentis* B8589 as starter cultures, exhibited significant changes during the initial time period (0–36 h). At the conclusion of fermentation, the levels of pyruvic acid, GABA, and capric acid exhibited a rise (9). In Supplementary Table 1 metabolomic and pathway analysis of various fermented milk products is shown during different conditions including storage.

Fermented milk is a significant fermented food that is widely acknowledged for its health benefits and its ability to serve as an effective vehicle for probiotics (35). *Streptococcus thermophilus* and *Lactobacillus delbrueckii* subsp. *bulgaricus* are the predominant primary bacterial cultures employed in conjunction with probiotics to facilitate the fermentation process of probiotic milk. Nevertheless, the simultaneous utilization of conventional starter bacteria and probiotics during fermentation poses a challenge in discerning the distinct biochemical impacts and metabolic processes attributed solely to probiotics (9). In a study, *Pediococcus pentosaceus* L1 and *Streptococcus thermophilus* L3 isolates from Laban, a traditional fermented milk showed high probiotic potential with promising EPS and antioxidants production (36). In another study, dual-strain-fermented milk (produced by both *Lacticaseibacillus paracasei* PC-01 and *Bifidobacterium adolescentis* B8589) was more stable and contained more beneficial amino acid metabolites (particularly GABA and L-malic acid) compared with the *Lacticaseibacillus paracasei* PC-01-fermented milk over 30-day storage (34). In a study assessed the antioxidant effects and probiotic content of different ratios of Rayeb milk and quinoa milk mixture; promising results were obtained and all Rayeb milk samples, particularly those that contained quinoa milk showed high *Lactobacillus acidophilus* and *Bifidobacterium bifidum* count as accepted probiotic (37). On the other hand, goat milk with or without fed with linseed (to enriched with omega-3 fatty acids) fermented with *Lacticaseibacillus paracasei* Shirota or *Lacticaseibacillus rhamnosus* A2 and *Lacticaseibacillus paracasei* FS109 showed high number of viable probiotic organism during storage (38). Adding a dehydrated cashew by-product to ferment with probiotic *Lacticaseibacillus paracasei* subsp. *paracasei* F19 and the starter *Streptococcus thermophilus* STM6 milk enhanced the phenolic content and thus antioxidant capacity (39). *Lactobacillus plantarum* 5H1 and *Lactobacillus plantarum* 5 L1, isolated from breast milk, demonstrated significant antibacterial activity, antimicrobial susceptibility, a wide range of enzymatic activity, adhesion to Caco-2 cells, and a reduction in *Salmonella enterica* adherence. Furthermore, these chosen strains exhibited notable vitality throughout fermentation and storage of fermented milk at 4 °C. These findings may facilitate the advancement of LAB fermented milks with probiotic attributes to enhance host health (40).

Milk-derived probiotics can be utilized for the production of various functional beverages. A study was conducted to evaluate the antioxidant and probiotic characteristics of oat and soy milk that were fermented using three distinct strains of *Lactiplantibacillus plantarum*, namely *Lactiplantibacillus plantarum* 12–3, *Lactiplantibacillus plantarum* K25, and *Lactiplantibacillus plantarum* YW11, which were obtained from Tibetan Kefir. The study’s findings indicate that soy milk and oat milk, when fermented with *Lactiplantibacillus plantarum* strains, exhibit favorable probiotic, antibacterial, and antioxidant characteristics (41). Evidence demonstrates that the fermentation of quinoa with *Lacticaseibacillus casei* enhances the nutritional value, bioactivity, and volatile components (42).

**Figure 1:**
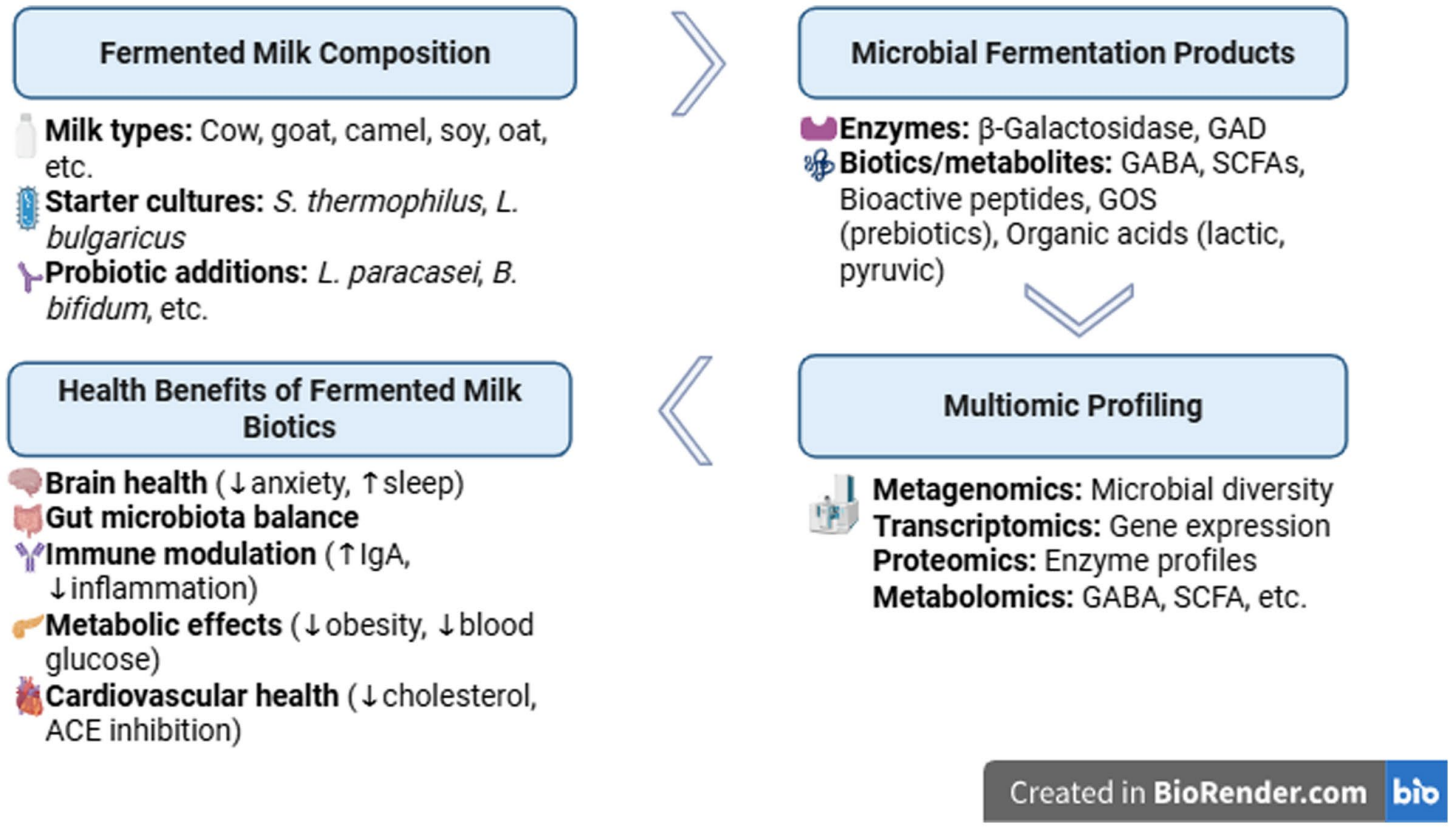
Multibiotics and potential health benefits of fermented milk.

During the fermentation process the interactions resulted in several multibiotics and multiomics that may be beneficial on human health (Figure 1). The *in vivo* study indicated that the probiotic milk fermented with *Lactobacillus helveticus* MTCC 5463, *Streptococcus thermophilus* MTCC 5462, and *Lacticaseibacillus rhamnosus* MTCC 5946 exerted a better anti-obesity effect (43). Additionally, a recent study showed postbiotics of *Lactobacillus helveticus* MTCC 5463 had anticancer effects (44). In Wistar-Kyoto rats fed with fermented milk containing the probiotic *Bifidobacterium animalis* BB-12 and pomegranate juice decreased the anxiety-related behaviors and increase the sleep quality through the gut-brain axis (45). Fermented milks containing *Limosilactobacillus* J20 and J23 that were shown as the most proteolytic strains, presented angiotensin converting enzyme, thrombin enzyme and micellar solubility of cholesterol inhibitory activities and potential cardio-protective effects (46). *Propionibacterium freudenreichii* CIRM-BIA129, a probiotic strain known for its anti-inflammatory properties,-fermented milk prevented colitis potentially with its fat content (47). *In vivo* analyses examined a fermented milk product made with four types of LAB revealed that the product effectively reduced allergy symptoms by regulating the immune response. Specifically, it modulated the balance between T helper cell (Th) 1/Th2 and Th17/T regulatory cell immune responses. Additionally, the product reduced levels of total IgG, total IgG1, and total IgE antibodies, as well as mast cell protease in the bloodstream, and histamine levels in both the bloodstream and the composition of the intestinal microbiota (48). Recent evidence suggests that fermented milks may contain certain bacteria and/or metabolites, such as peptides, EPS, free amino acids, organic acids, and vitamins (49). These compounds are released during fermentation and have the ability to reduce the production of pro-inflammatory cytokines associated with Th17 cells, including IL-17, IL-10, IFN, IL-6, IL-23, and TGF-β. This is achieved by influencing TLR signaling and the differentiation of native Th cells into Th1, Th2, or Th17 effector cells. Therefore, these fermented milks offer a hopeful alternative for the management of inflammatory bowel disease (49).

The diverse range of food compositions, fermentation microorganisms, and techniques employed, coupled with limited understanding of the composition and properties of many traditional fermented goods, pose a difficulty in attributing a specific health advantage to a particular constituent or constituents of a fermented food. The positive effects of fermented foods are generally linked to the presence of many bioactive substances; including phenolic substances, prebiotic-like substrates, and the microorganism engaged in the fermentation process, in addition to the macro- and micro-nutrients present (10). Dairy starter cultures have the ability to produce lactic acid, peptides, vitamins, extracellular polysaccharides, and other compounds through their metabolic processes, given the right conditions. LAB metabolizes lactose in milk, transforming it into lactic acid, while proteins are broken down into polypeptides or amino acids. The fermentation process generates organic acids, alcohols, esters, ketones, and other flavor compounds, which provide fermented milk distinct flavors and abundant nutrients (50).

Galactooligosaccharides (GOS) are substances present in fermented products. They are generated through the activity of glycoside hydrolase enzymes, such as β-galactosidase, which utilize lactose as a substrate. Lactose is the primary sugar found in milk. These enzymes are synthesized by several bacteria, including *Bifidobacterium adolescentis*, *Bifidobacterium bifidum*, *Bacillus* spp., *Streptococcus thermophilus*, and *Lactobacillus acidophilus*, several fungi, like *Kluyveromyces lactis*, have the ability to create glycoside hydrolases. These enzymes are present in several fermented milk products that have been discussed in this study. GOS are well recognized as prebiotics due to their ability to influence the functions and structure of the gut microbiota (26). Moreover, they improve lactose digestion by enhancing the activity of the β-galactosidase enzyme, resulting in specific health benefits. Lactose from milk is broken down by β-galactosidase into glucose and galactose, which is then taken up by enterocytes and utilized as an energy source. *Lactiplantibacillus plantarum, Limosilactobacillus fermentum, Lactiplantibacillus pentosus, and Lactiplantibacillus* sp. have been identified as highly potential probiotics capable of generating β-galactosidase (51).

During the process of milk fermentation, LAB is also accountable for the synthesis of many secondary metabolites, including volatile compounds, peptides, and organic acids. These molecules have diverse effects on the technical, nutritional, and sensory characteristics of the resulting products. GABA has garnered significant interest among the bioactive chemicals that have been released. The LAB species *Levilactobacillus brevis, Lacticaseibacillus paracasei, Lactiplantibacillus plantarum,* and *Lactococcus lactis* are capable of producing GABA by the enzymatic reaction of glutamate decarboxylase, which involves the *α*-decarboxylation of glutamate. This reaction is facilitated by the pyridoxal 5′-phosphate dependent enzyme (18, 52). A study shown that the GABA-producing capacity of two strains of *Lactococcus lactis* in milk was increased by the addition of monosodium glutamate and by co-culturing with either *Lacticaseibacillus rhamnosus* or *Lacticaseibacillus paracasei* (52). Recently, consumption of GABA added and high GABA producer *Levilactobacillus brevis* DL1-11 fermented milk showed improvements on insomnia by modulating gut microbiome and increased SCFAs (53).

## 3 Yogurt

The Codex Alimentarius defines yogurt under the Codex Standard for Fermented Milks (CODEX STAN 243–2003). Yogurt (or yoghurt) is produced by the fermentation of milk using a symbiotic culture of two LAB: *Streptococcus thermophilus* and *Lactobacillus delbrueckii* subsp. *bulgaricus*. These microorganisms must remain viable, active, and present in sufficient numbers, with the total count of the starter culture microorganisms being at least 10^7^ colony-forming units (cfu) per gram in the final product, up to the date of minimum durability (54).

The fermentation of milk to yogurt attributes to its formation of flavor, texture, added functionality, and nutritional value. Through fermentation, *Lactobacillus* species generate a diverse range of metabolites. At the molecular level, the yogurt matrix is composed of hundreds of compounds, including proteins, lipids, carbohydrates, and various metabolites such as amino acids, organic acids, bioactive peptides, nucleic acids, fatty acids, minerals, and aroma volatiles, which contribute to its flavor characteristics (55).

Yogurt compounds and metabolites may vary depending on the type and the content of the milk used in production, which is affected by various determinants such as genetics, seasonal changes, lactation, feed, etc. (56). Additionally, the composition and nutritional value of the final yogurt product are impacted by processing methods, including thermal treatments and fermentation, as well as factors such as the type of bacteria, fermentation temperature, and time. Also, potential contamination, spoilage, or microbial activity during the fermentation can alter the metabolite profile. Therefore chemical composition of yogurt can be used to predict its nutritional quality, safety, and sensory (57, 58).

The composition of metabolites may vary depending on the raw materials utilized. Distinct metabolite profiles were identified between cow milk yogurt (CY) and goat milk yogurt (GY) through GC–MS-based untargeted metabolomics. The increased levels of free amino acids and dipeptides in GY suggest enhanced proteolytic activity on goat milk proteins by bacteria, whereas the elevated tri-peptide levels in CY indicate superior texture. GY’s abundance of medium-chain fatty acids led to increased levels of carboxylic acids and fatty acid derivatives. Furthermore, the upregulation of intercellular signaling molecules in GY suggests pH regulation during storage. It has also been shown that storage duration had a significant effect on metabolites (59). In another study, the discriminant analysis results showed significant differences in the metabolite profiles analyzed using GC–MS between the two yogurt made from sheep milk and goat milk processed with the same manufacturing procedures. Goat milk yogurt exhibited higher concentrations of free amino acids, γ-aminobutyric acid, pyroglutamic acid, and β-phenyllactic acid compared to sheep milk yogurt. Conversely, sheep milk yogurt was characterized by elevated levels of myoinositol, N-acetylgalactosamine, and N-acetylglucosamine (60).

Trimigno et al. (61) conducted an NMR-based metabolomics study, which revealed that the choice of starter cultures (*Streptococcus sali*var*ius* subsp. *thermophilus*, *Lactobacillus delbrueckii* subsp. *bulgaricus*, or their combination), along with the application of different heat treatments to milk (99 °C or 105 °C), resulted in distinct variations in the metabolite profile during the yogurt fermentation process. The study reveals a notable breakdown of proteins and lactose during fermentation, alongside a concurrent rise in acetate, lactate, and citrate levels. Formate concentrations varied based on milk heat treatment, with their trajectory influenced by starter cultures. Lactobacillus requires formate for growth but cannot produce it, while Streptococcus can generate formate from pyruvate, fostering a symbiotic relationship, and Lactobacillus enriches the final product by hydrolyzing milk proteins into amino acids. Another study showed that fermentation with a starter culture increased peptide abundance in dairy products, as shown by LC–MS and NMR. Fermented yogurts showed increased peptide abundance, enhanced bioactive potential, and distinct profiles of peptides, amino acids, and small compounds compared to chemically acidified yogurts and milk. Post-fermentation activity during 14 days of cold storage further increased peptide levels. Heat inactivation altered peptide profiles but maintained or even increased peptide content, while fermentation reduced lactose and increased galactose and organic acids, indicating proteolysis and saccharolytic activity (55).

The dairy industry is currently focusing on developing innovative functional products by incorporating probiotic bacteria into fermented milk (16). Probiotic yogurt contains added live beneficial bacteria in addition to the traditional yogurt starter cultures, which may improve its nutritional value. Probiotic yogurt products typically contain various strains of *Lactobacilli* and *Bifidobacteria* (62).

During fermentation, factors such as pH, temperature, aeration, standardization, and composition of fermentation cultures play significant roles in influencing the growth of probiotic bacteria. Some studies suggest that fermentation with probiotics leads to distinct metabolomic profiles compared to fermentation without probiotics, as probiotic bacteria can also influence the formation of volatile metabolites (63). Wang et al. (64) investigated the impact of different fermentation temperatures on the growth behaviors, as well as the volatile metabolomic profiles of yogurts using multistrain probiotics of *Lacticaseibacillus casei* Zhang (LCZ) and *Bifidobacterium lactis* V9 (*V9*). LC–MS and GC–MS were employed for analyzing and comparing the growth behaviors and metabolomic profiles of yogurts. The study revealed that the addition of LCZ led to significant alterations in nonvolatile metabolomic profiles, such as increased levels of galactose, amino and nucleotide sugars, fructose and mannose, purine, Phe metabolism, and Arg biosynthesis. Multistrain probiotics had a higher contribution to the changes in volatile and nonvolatile metabolomic profiles at 42 °C than those at 37 °C.

There has been a growing interest in the effects of yogurt type and added sweeteners on fecal short-chain fatty acids (SCFAs) and gut microbiota. In a human study, there was no significant difference observed between the fecal SCFA amounts of those consuming yogurt, fermented milk, and sweetened yogurt. However, sweetened yogurt consumers displayed significantly lower fecal levels of *Bacteroides* than non-consumers (65). Consumption of certain sweeteners like sucralose may decrease the amount of *Bacteroides* in the gut (66).

Omics technologies contribute to understanding how functional ingredients, such as lactosucrose (LS), affect the metabolome. LS is known for its prebiotic effects and its ability to improve intestinal mineral absorption (67). One study utilized untargeted metabolomics through UPLC Q-TOF MS/MS to explore the impact of LS-enriched yogurt on metabolite production and related metabolic pathways. The results revealed 45 notable metabolites involved in amino acid, thiamine, nicotinic acid, and pyrimidine metabolism. In particular, levels of L-arginine, L-proline, and L-glutamic acid were elevated, whereas glutathione, L-tyrosine, and L-phenylalanyl-L-proline levels were reduced. These findings suggest that incorporating LS can enhance the formation of metabolites, offering potential for innovation in functional dairy products (68).

Components of yogurt are suggested to play a positive role in the prevention of NCDs (69). Therefore, identifying the differences in matrix characteristics of yogurt is important to deeply understand this effect. As cheeses and yogurts have shown more enhanced health benefits than regular milk, it has been proposed that fermentation plays a role—highlighting the need for dietary guidelines to differentiate between fermented and non-fermented dairy products and further exploring the underlying metabolic mechanisms (14).

Different types of dairy products may elicit distinct postprandial metabolomic profiles, with the resulting metabolites potentially exerting diverse physiological effects. Bütikofer et al. (70) identified lactose and its derivatives as potential indicators of lactose-containing dairy intake, with their profiles modulated by whether the product was fermented. Indole-3-lactic acid and 3-phenyllactic acid, both products of fermentation, clearly distinguished yogurt from milk. Moreover, fermentation of milk was found to enhance the immediate availability of free amino acids in humans. The compound 3,5-dimethyloctan-2-one was also identified as a distinguishing marker for both milk and yogurt intake. In a randomized controlled trial involving 48 obese women with metabolic syndrome and nonalcoholic fatty liver disease, either yogurt or milk (220 g/day) consumption for 24 weeks showed significantly different metabolic profiles at the end of the intervention with untargeted metabolomics analysis using NMR and UPLC-Q-TOF-MS. These findings highlight the potential mechanisms through which yogurt may mitigate metabolic disorders, providing insight into the integrated metabolic alterations induced by yogurt consumption (71). However, in a different study where participants consumed 400 g/day of milk, yogurt, heat-treated yogurt, or chemically acidified milk as part of their regular diet for 16 weeks, NMR-based metabolomics was applied to plasma, urine, and fecal samples collected before and after the intervention. The only notable change in the plasma metabolome was an increase in citrate levels associated with yogurt consumption. No significant differences were observed in the urine metabolome. In contrast, both acidified milk and heat-treated yogurt led to alterations in the fecal metabolome, including reduced levels of certain amino acids (leucine, valine, and threonine) and branched-chain fatty acids (BCFAs) (72). In another randomized controlled crossover trial, serum samples were analyzed following the acute intake of milk versus yogurt. Both untargeted (LC–MS) and targeted (GC–MS) metabolomics approaches were used to assess serum profiles. Results showed elevated levels of lactose, galactonate, and galactitol after milk consumption, while yogurt intake led to a higher concentration of 3-phenyllactic acid—a compound generally regarded as a biomarker of fermented foods (73).

While the positive effects of yogurt on metabolic health are well-documented, few studies have thoroughly explored the comprehensive metabolic changes induced by yogurt consumption. Further research is needed to fully understand the metabolic alterations associated with yogurt intake.

## 4 Sour cream

Sour cream is a fermented milk product created by souring pasteurized cream by lactic acid-producing bacteria (74). It has a milky white color, pleasant smell and delicious taste. It can be eaten directly with bread and used as a condiment in baking various pastries (75). It can be added to potatoes, salmon and salad (76).

At the industrial scale, mixed mesophilic LAB strains are employed as starter cultures in sour cream production, predominantly comprising *Lactococcus lactis* subsp. *lactis*, *Lactococcus lactis* subsp. *cremoris*, *Lactococcus lactis* subsp. *lactis* biovar *diacetylactis*, *Leuconostoc mesenteroides* subsp. *cremoris*, and *Leuconostoc citrovorum* (77). For this reason, sour cream contains microbial biodiversity rich in LAB. In a study, 14 bacterial phyla, 155 bacterial genera and 267 different bacterial species were found for sour cream. The major bacterial phyla (average relative abundance >1%) were identified as Firmicutes (81.47%), Proteobacteria (16.11%) and Bacteroidetes (2.05%). The most common genera are *Lactococcus* (48.21%), *Streptococcus* (23.63%), *Curvibacter* (8.15%), *Acinetobacter* (5.43%), *Lactobacillus* (2.21%), *Chryseobacterium* (1.90%), *Pseudomonas* (1.71%) and *Carnobacterium* (1.13%). The most abundant species are *Lactococcus lactis* (39.39%), *Streptococcus thermophilus* (10.60%), *Lactococcus raffinolactis* (6.11%), *Acinetobacter lwoffii* (3.07%), *Lactococcus chungangensis* (1.76%), *Acetobacter cibinongensis* (7.72%) and *Acinetobacter johnsonii* (1.38%) (75). Similarly, in another study, *Leuconostoc mesenteroides, Lactococcus lactis, Lactiplantibacillus plantarum* and *Lactobacillus helveticus* strains were reported to be the dominant isolates, respectively (78). *Streptococcus thermophilus* and *Lactococcus lactis*, usually isolated from fermented milk products, are known as the most important microorganisms in the dairy industry. It has been reported that *Lactococcus raffinolactis* combined with *Lactococcus lactis* can be used as a new starter culture in fermented milk due to their strong complementarity during the fermentation process (79, 80).

There are several commercially available postbiotics derived from LAB that play significant roles in immunomodulation, gut dysbiosis, and intestinal disorders. These include Aktoflor-S and C, CytoFlora, Del Immue V, Zakofalk and Hylak forte. Probiotic fermentates obtained from *Lacticaseibacillus rhamnosus* and *Propionibacterium jensenii* have been reported to be used as antifungal agents in sour cream (81).

Our knowledge of the metabolites present in traditionally fermented sour cream is limited. During fermentation, which is a metabolic process, different chemicals are released as metabolites. The concentration of these metabolites may change over time, from production to consumption of fermented food, due to the activity of microorganisms. This phenomenon has an impact on the quality of fermented foods. As a result, there is increasing emphasis on research aimed at identifying the metabolic profiles and microbial communities present in fermented foods (82, 83).

The incorporation of probiotic microorganisms into dairy products can lead to alterations in their physicochemical and rheological characteristics. During fermentation, probiotics may influence the bioavailability of fatty acids, thereby modifying the fatty acid composition of milk (84). Specifically, certain probiotic strains have been shown to enhance the proportion of unsaturated fatty acids and extend the chain length of medium-chain fatty acids in cream. The degree of fatty acid saturation can vary depending on the specific probiotic strain utilized. Consequently, the development of probiotic-enriched dairy products holds significant value not only due to their functional benefits – such as the presence of live beneficial microorganisms and naturally occurring prebiotics – but also for their potential to reduce the fat content in cream (85).

Valuable insights have been provided into the metabolite profile of sour cream. In a study, 19 main metabolites (Tyr-cys, Uridine, His-asn, Lys-val, Decanoic acid, Met-tyr, Isohexanal, Lys-lys, Palmitic acid, 2’-Deoxycytidine, L-Xylulose, Leu-val, Serine, L-Cysteine, Cystine, Aspartic acid, L-Lysine, L-Alanine, gly-ser-pro-met-phe-ala-val) for sour cream were identified by omics techniques. The most dominant metabolites are Lys-lys, Isohexanal, Palmitic acid, Leu-val and 20’-Deoxycytidine (75). Moreover, no proteomic or lipidomic studies about sour cream have been found in the literature, as far as we know.

A study examining the impact of meals with equivalent fat content from various dairy products on TG concentrations over a 6-h period in healthy adults revealed that sour cream led to a 53% higher increase in TG levels compared to butter, and a 23% higher increase compared to cheese. Nevertheless, the underlying mechanisms and their potential clinical relevance remain to be fully understood (86). Given that fermented foods have been linked to either neutral or positive outcomes in terms of cardiovascular health, it is reasonable to anticipate that sour cream, as a fermented dairy product, may exhibit similar beneficial effects. However, the effect of sour cream on TG concentrations at 6 h after the meal may suggest that sour cream may be more atherogenic. Nonetheless, the same study reported a peak in TG levels 2 h following sour cream consumption. Notably, evidence indicates that postprandial TG levels (i.e., assessed within 8 h following meal consumption) serve as a more reliable predictor of cardiovascular disease risk compared to fasting triglyceride concentrations (86, 87). Postprandial levels of HDL cholesterol – which is inversely correlated with the risk of cardiovascular events – are generally anticipated to remain stable or decrease slightly following food intake (88). With the increase in omics studies, the mechanisms of sour cream’s effect on health can be better elucidated.

## 5 Buttermilk

Buttermilk is often used in the food industry and as animal feed. It is also added to the formulation of conventional dairy products (89). Buttermilk can be produced by two principal methods: cultured buttermilk, obtained through the lactic acid–mediated acidification of cream, and sweet buttermilk, the aqueous fraction released when cream is mechanically churned into butter (90).

Cultured buttermilk is produced by adding commercial strains (e.g., *Lactococcus lactis* ssp. *lactis, Lactococcus lactis* ssp. *cremoris, Leuconostoc mesenteroides* ssp. *cremoris, Streptococcus lactis, Lactococcus lactis* ssp. *lactis* biovar). *Lactobacillus helveticus* followed by *Lactobacillus kefiranofaciens*, *Lactobacillus delbrueckii* and *Lactobacillus kefiri* are the LAB species commonly found in buttermilk. As a result of pyrosequence analysis of rDNA amplicons, microorganisms such as *Methylobacterium populi, Methylobacterium radiotolerans, Ralstonia solanacearum, Synechocystis* sp. and *Thermoanaerobacter* sp., which have not been associated with food fermentation before, were also revealed (12).

Buttermilk, which is a natural combination of bioactive proteins and small lipids, is similar to skim milk except that it contains a higher percentage of milk fat globule membrane (MFGM) (91). The isolation and purification processes of buttermilk MFGM components have been examined in various studies in recent years. In this context, a detailed understanding of the lipid and protein composition of the MFGM is essential to fully elucidate the functional and nutritional potential of buttermilk (90, 92–94).

The lipid fraction of the MFGM is predominantly composed of neutral lipids (approximately 70%), including triglycerides, diglycerides, monoglycerides, cholesterol esters, and free cholesterol. The remaining portion primarily consists of polar lipids, such as phospholipids (around 30% – notably phosphatidylethanolamine, phosphatidylcholine, phosphatidylserine, and phosphatidylinositol) and sphingolipids, with sphingomyelin being the most abundant. Additionally, minor components like gangliosides and free fatty acids may also be present (91, 94). Buttermilk contains approximately six times the polar lipid content of whole milk, making it a highly suitable source of MFGM (89). Approximately 20% of the proteins in buttermilk are derived from the MFGM. Buttermilk is particularly rich in MFGM proteins, including butyrophilin (BTN), xanthine oxidase/dehydrogenase (XO/XDH), lactadherin (PAS6/7), adipophilin (ADPH), and fatty acid-binding protein (FABP). Among these, BTN is the most prevalent, constituting about 40% of the total MFGM protein content (90, 95). Due to its nutritional composition, buttermilk has garnered growing interest in recent years and is recognized as a functional food (96).

While the MFGM proteins of buttermilk, one of the main by-products of the dairy industry, are higher than those of the by-products obtained during cheese and milk cream making; The MFGM oils of the products obtained during cheese and milk cream making are higher than buttermilk. The properties of these components can vary considerably based on the processing conditions of the raw materials as well as the methods used for the isolation and purification of the MFGM (94). Depending on the context, many health benefits of proteomics and lipidomics can be mentioned. For instance, the beneficial impacts of consistent MFGM consumption on neurological and cognitive development, along with immune and gastrointestinal health, have been well-documented. Additionally, MFGM has been shown to enhance insulin sensitivity and decrease inflammatory markers, LDL-cholesterol, and triglyceride levels by inhibiting intestinal cholesterol absorption and promoting its excretion through feces (97). This effect was thought to be likely due to the ability of polar lipids from MFGM to inhibit cholesterol micelle solubility (98). In a study by Conway et al., it was observed that the daily intake of 45 grams of non-fermented buttermilk over a 28-day period by healthy individuals resulted in a 10.7% reduction in triacylglycerol levels and a 3.1% decrease in total serum cholesterol. While the reduction in serum triacylglycerol was considered practically significant, the influence of buttermilk consumption on serum apoB100 levels did not reach statistical significance (99). In a study utilizing 80 mL of buttermilk and 1.5 egg yolks, it was found that buttermilk effectively mitigated the increase in serum LDL-cholesterol levels induced by egg yolk consumption. Additionally, buttermilk appeared to lower triacylglycerol and total serum cholesterol levels. Since no significant changes were observed in HDL- or LDL-cholesterol levels, the reduction in total cholesterol was attributed to a decrease in VLDL-cholesterol (100). It is stated that in those who consume buttermilk regularly, aging will slow down and arteries remain flexible for longer (101). A study investigating buttermilk derived from both washed and unwashed cream – processed via centrifugation or gravity – demonstrated that these samples inhibited the proliferation of SW480 colon cancer cells *in vitro* in a dose-dependent fashion. The antiproliferative effects were notably selective toward malignant cells. A microfiltered fraction enriched with lactosylceramide (44.3%) was shown to induce caspase-independent cell death, indicated by externalization of phosphatidylserine, elevated DNA fragmentation, and a reduction in mitochondrial membrane potential in SW480 cells. Furthermore, this fraction was found to suppress key signaling pathways associated with cell proliferation and survival, including those mediated by β-catenin, phosphorylated ERK1/2, Akt, and the oncogene c-Myc (96, 102). In a study investigating the effect of buttermilk consumption on blood pressure in moderately hypercholesterolemic individuals, 45 g/day buttermilk consumption for 4 weeks reduced systolic blood pressure by 2.6 mmHg, mean arterial blood pressure by 1.7 mmHg, and plasma levels of angiotensin I converting enzyme by 10.9%. However, no effect was found on plasma concentrations of angiotensin II and aldosterone (103). Nevertheless, additional studies are required to gain a deeper understanding of the mechanisms through which buttermilk influences human health and to explore its potential efficacy in the treatment and prevention of various diseases.

Metabolomics is one of the important and new technologies that attracts as much attention as other “-omic” studies. However, no studies on buttermilk have been found in the literature. Given the complex biochemical composition of buttermilk—rich in bioactive lipids, proteins, and membrane components—metabolomic approaches could provide valuable insights into its nutritional functionality, processing effects, and potential health benefits. The absence of such studies highlights a significant research gap that warrants further investigation (104).

## 6 Kefir

Kefir is a fermented beverage that can traditionally be produced in two different ways: milk kefir and water kefir. While water kefir constitutes an essential source of probiotics and prebiotics, especially for vegansor people with milk allergy/intolerance, milk kefir, which has similar probiotic and prebiotic content, also contains high protein (105).

Kefir is traditionally prepared by fermenting kefir grains in milk. Kefir grains are white-cream in color and come in tiny granules. Typically, cow’s milk is used to make kefir. Goat, sheep, camel, buffalo, donkey, whey, and herbal sources such soy, rice, coconut, and hazelnut milk can also be used to make milk kefir (106, 107). Kefir can be obtained by using pasteurized full-fat, semi-skimmed, or skim milk kefir production is traditionally carried out by adding kefir grains to pasteurized milk. This mixture is kept at 20–25 °C for 18–24 h until the pH 4.6. As a result, kefir grains increase their biomass by 5–7% and there is a dairy product with viscous, opaque, slightly acidic taste due to CO_2_ (105, 108).

LAB, yeasts, fungus, and acetic acid bacteria dwell in a symbiotic relationship within the natural matrix of kefir grains, which have a gelatinous structure and are composed of proteins, kefiran, and EPS (109). Kefir grains are made up of 86% water and 14% solid material. While this can differ based on the source of the kefir grains, the composition of the dry matter typically includes around 58% polysaccharides, 30% proteins, 7% fats, and 5% minerals (105).

The microorganism content in kefir varies depending on the kefir grains used and fermentation conditions. Significant differences can be observed in the content of kefir, especially as a result of traditional and commercial production of kefir. Traditional kefirs usually show considerable differences from commercial kefir in terms of microbial composition and metabolite profiles, whereas commercial kefir is typically made using planktonic cells. In contrast, traditional kefir fermentation begins with the kefir grain, which is a biofilm that is associated with the surface. Commercial cultures have lower microbial diversity and rarely contain yeast species. Conversely, conventional kefir typically has elevated levels of *Lactobacillus kefiranofaciens* or various specific lactobacilli found in kefir. Furthermore, owing to the diverse range of yeast species present, it may have increased concentrations of alcohol and esters when compared to store-bought kefir (110). It is seen that the amount of LAB is more dominant than acetic acid bacteria in kefir. For example, while *Lactobacillus kefiranofaciens* is dominant at the beginning of fermentation*, Leuconostoc mesenteroides* may dominate at the end (111).

Another factor that affects the content of kefir is the type of milk used. For example, when the microorganism contents of kefir made with goat, sheep, and cow milk were examined, it was seen that the lactic acid and acetic acid bacteria and yeast contents in sheep milk were higher than in goat and cow milk (112).

Kefir grains are approximately 50% *Lactobacillus* spp., 20% *Leuconostoc* spp., 10% *Streptococcus* spp., 8% *Pediococcus* spp., 7% *Lactococcus* spp., and 5% other types of bacteria (113). In general, *Lactobacillus kefir, Lactobacillus kefiranofaciens, Lactobacillus kefirgranum, Lentilactobacillus parakefiri, Lacticaseibacillus paracasei, Lactiplantibacillus plantarum, Lactobacillus acidophilus, Levilactobacillus brevis, Lactobacillus helveticus, Leuconostoc mesenteroides, Kluyveromyces lactis, Lactobacillus delbrueckii* subsp. *bulgaricus*, *Saccharomyces cerevisiae, Saccharomyces unisporus,* and *Candida kefir* contents of milk kefir grains are dominant (105, 114). Despite these microorganisms being dominant in the content of kefir grains, the microorganism content of the final product may not be the same as the grains. The total LAB content of kefirs, especially those fermented in milk-based environments, is known to be higher (115). The microorganism content of kefir detected in some studies is shown in Table 1.

**Table 1:** Microorganism content of kefir shown in the literature.

Production of kefir	Microorganism content		
	Phylum and/or family	Genus or species	Yeast (genus or species)
It was prepared by mixing pasteurized whole milk with kefir grains in a ratio of 1:10 at 25 ± 2 °C in 24 h (197).	Phylum 65% Firmicutes 31.1% Bacteroidetes 4.8% Proteobacteria Family 61.2% *Streptococcaceae* 2% *Lactobacillaceae*	62% *Lactococcus* 10.6% *Bacteroides* 0.2% *Pseudomonas* 0.1% *Rahnella*	71.9% *Aspergillus* 16.9% *Cordyceps bassiana* 5.1% *Saccharomyces* 2.1% *Sarocladium* 2% *Cladosporium* 1% *Fusarium*
It was prepared by mixing UHT full-fat cow’s milk with kefir grains in a ratio of 1:10 at 25 ± 2 °C in 24 h (126).	Phylum 81.3% Proteobacteria 17.1% Firmicutes 1.6% Bacteroidetes Family 54% *Burkholderiaceae* 16.2% *Enterobacteriaceae* 11.7% *Carnobacteriaceae* 8.3% *Pseudomonadaceae* 3.4% *Enterococcaceae* 2.9% *Moraxellaceae* 1.9% *Staphylococcaceae* 1.6% *Bacteroidaceae*	54% *Comamonas* 13.2% *Hafnia-Obesumbacterium* 11.7% *Carnobacterium* 8.3% *Pseudomonas* 3.4% *Enterococcus* 2.9% *Acinetobacter* 1.9% *Staphylococcus* 1.6% *Serratia* 1.6% *Bacteroides* 1.4% *Buttiauxella*	
Commercial product (136)	First kefir sample 51.72% Actinobacteria 23% Proteobacteria 21.5% Firmicutes Second kefir sample 45.5% Actinobacteria 14.28% Firmicutes 11.67% Proteobacteria	First kefir sample 28% *Lactobacillus* Second kefir sample 18% *Lactobacillus*	First kefir sample 3% *Saccharomyces* Second kefir sample 30% *Saccharomyces*
Prepared with sterilized skim milk at 26 °C for 8, 24, 36 h (120).	97% *Lactobacillaceae*	86.02% *Lactobacillus kefiranofaciens* 3.44% *Lactobacillus helveticus* 2.45% *Lentilactobacillus parakefiri* 1.32% *Lactobacillus crispatus*	
It was prepared by mixing pasteurized whole milk with 5% kefir grains at 22 °C in 24 h (133).		98.8% *Lactobacillus* 0.7% *Lactococcus* 0.3% *Leuconostoc* 0.2% *Enterococcus*	70.4% *Kluyveromyces* 29.2% *Saccharomyces* 0.3% *Torulaspora* 0.1% *Kazachstania*

The geography where milk kefir first appeared is considered to be the North Caucasus mountains in Russia. It is known that the first kefir was made with fresh milk fermented in bags made of goat skin and was known as the secret of long life among the people of the region. Since then, kefir, whose health effects have become more prominent, has become more popular and its consumption has increased day by day, is produced with much more modern technological methods today (116).

Various metabolites and microorganisms obtained during fermentation form the basis of kefir’s positive health effects. The digestive system benefits from probiotics and prebiotics such GOS and their extracellular enzymes, which are found in kefir. Antibacterial, antioxidant, antihypertensive, anti-inflammatory, anticancer, antidiabetic, antiallergic, and cholesterol-lowering properties are among the health advantages of kefir that have been reported. Other benefits include effects on the immune system, digestive system, lactose intolerance, neurological diseases, and sleeplessness (105). Studies on rats have shown the positive effects of kefir on obesity, hyperlipidemia, and hyperglycemia (117), as well as its effects on a carcinogenic fungus (118). It has also been shown that kefiran, the EPS of kefir, has fundamental physicochemical properties and biological activities (119).

Although the microbial compositions of kefir vary significantly after fermentation with kefir grains, the core microbiome and metabolite profile are relatively consistent (120). Carbon dioxide, lactic acid, acetaldehyde, acetoin, and a trace amount of ethanol are generated while kefir ferments. Furthermore, during kefir fermentation, peptides, polysaccharides, polyphenols, amino acids, and other bioactive components with a variety of nutraceutical benefits are generated, along with more than 50 distinct fragrance compounds (121, 122).

Fermentation of kefir generally promotes the breakdown of macromolecules in milk and their transformation into functional substances. After fermentation, although it varies depending on many conditions related to fermentation, an average of 700 metabolites were detected in kefir. Nucleosides, nucleotides, and analogs (2.02%), alkaloids and derivatives (0.60%), organic nitrogen compounds (1.41%), organic polymers (0.4%), benzenoids (7.66%), lipids and lipid-like molecules (44.56%), organic acids (14.52%), organoheterocyclic compounds (11.90%), organic oxygen compounds (9.88%), phenylpropanoids and polyketides (6.65%), and hydrocarbons (0.4%) are among these metabolites (120). Especially in traditionally produced kefirs, it is seen that polyesters, glucose, and ethanol are high due to the high yeast activity. It is observed that organic acid and aldehyde levels are high in kefirs with high levels of lactobacilli, such as *Lactobacillus kefiranofaciens*, which promote proteolysis (123).

One of the most important metabolites of kefir is kefiran, an EPS. Kefiran production is directly related to *Lactobacillus kefiranofaciens*. The biological characteristics of this EPS, including its prebiotic, antioxidant, antibacterial, anticancer, and neuroprotective activities, draw interest (124). On the other hand, it has been shown that the EPS structures of kefir are also associated with *Acidaminococcus timonensis*, and these compounds have anti-inflammatory activity in the intestine. It is also stated that microorganisms such as *Anaerostipes butyraticus*, *Roseburia faecis*, and *Roseburia hominis* in kefir play a role in producing short-chain fatty acids (125).

Differential metabolites during fermentation are used to identify pathways involved in lipid metabolism. Lipid composition has been found to be significantly impacted by *Lactobacillus plantarum*. It has been observed that glycerophospholipids and sphingolipids generally increase and fatty acids decrease during fermentation. It is thought that this will increase the anti-inflammatory effect (120). Kefir promotes the production of catalase and superoxide dismutase in the colon as well as short-chain fatty acids (butyrate) in the brain and feces (butyrate and propionate). It is also known to lower triglycerides and uric acid and to influence the microbiota’s fecal butyrate-producing bacteria (*Lachnospiraceae* and *Lachnoclostridium*). Butyrate is associated with reducing DNA damage in intestinal colonocytes. All these effects show that kefir metabolites positively affect intestinal and brain health (126).

Amino acids and polypeptides are prevalent during the initial stages of fermentation (127). The formation of secondary metabolites, such as alkaloids, glucosinolate, phenylpropanoids, and folate, also requires an early stage (128). The pentose phosphate pathway, galactose metabolism, and the metabolism of amino sugars and nucleotide sugars are observed to be active by the middle of fermentation. While galactose and glucose can be used by most yeasts for fermentation, lactose cannot be used directly. LAB have peaked and are now able to break down lactose into galactose and glucose, which gives the yeasts a carbon supply (129).

The cell membrane proteinases secreted by *Lactobacillus* hydrolyze proteins into short peptides and amino acids. Tyrosine, tryptophan, phenylalanine, valine, leucine, and isoleucine are the primary metabolites involved in this process (130). Peptides and amino acids released during these events are responsible for a significant part of the positive effects of kefir, such as blood pressure regulation and antibacterial and antioxidant effects. For instance, a variety of physiologically active peptides, such as ACE inhibitor peptides, are produced by the symbiotic metabolic processes of certain bacterial and yeast species in kefir, which include the proteolytic and lipolytic breakdown of milk components (131, 132). Angiotensin-converting enzyme (ACE) angiotensin I is inhibited by ACE inhibitors from becoming the powerful vasoconstrictor angiotensin II. Consequently, it prevents the generation of aldosterone. Bradykinin, a vasodilating hormone that encourages an increase in serum sodium concentration, is broken down by the hormone aldosterone, which influences both blood pressure increases and decreases (108). Furthermore, in mice given high fructose corn syrup, peptides extracted from kefir were found to enhance triglyceride accumulation, tumor necrosis factor-*α* (TNF-α) and interleukin-1β (IL-1β) levels, and SREBP-1c expression in the liver. Kefir consumption has been demonstrated to decrease the liver’s expression of SREBP-1c and FASN mRNAs in high fructose-fed rats. It has also been reported to decrease the accumulation of macrovesicular fat in addition to the expression of the SREBP1 and FASN genes. In other words, in rats fed high fructose, kefir inhibits both inflammation and hepatic lipogenesis at the same time (133). Furthermore, it has been demonstrated that kefir lowers the levels of the liver enzymes ALT and AST (126).

Kefir was found to raise the penos, penos/total eNOS ratio, and the gene and protein expressions of IRS-1 in rats’ livers in relation to insulin resistance. Thus, it has been demonstrated that kefir has a moderately mending effect on the liver’s insulin signaling pathway. Furthermore, following kefir application in the liver, a decrease in the gene expression levels of the hepatic fructose transporters GLUT2 and GLUT5 was found (133).

Early in the fermentation process, aconitic acid expression is higher. The production of secondary metabolites, such as glucosinolate, alkaloids, and phenylpropanoids, which are produced from the biosynthesis of phenylalanine and folate, is also seen to be intense at this stage (128). Alkaloids are one of the compounds that contain nitrogen and have many direct pharmacological effects. Anionic natural chemicals high in sulfur and aromatic molecules, glucosinolates have biological properties like antibacterial and anticancer properties (120). Furthermore, kefir metabolites cause a rise in immune system cells like IgA and stimulate apoptotic cell lysis in tumors, which dramatically inhibits tumor growth (134).

In addition to the metabolites released during the fermentation of kefir, the types of microorganisms it contains also have activities in the host. For instance, kefir contains a type of bacterium called *Comamonas*, which is well-known for its diverse catabolic properties. Numerous organic substrates, including as amino acids, carboxylic acids, steroids, and aromatic compounds, can be catabolized by this species (126). However, LAB like *Enterococcus* and *Carnobacterium* in kefir can produce EPS that can boost colonic fermentation and the rate of *Comamonas* genus microorganisms while significantly lowering the rate of pathogenic enterobacteria like *Shigella*, *Escherichia coli*, and *Helicobacter* (129).

Kefir grains may potentially improve intestinal health by influencing the bacterial population, which has been shown to modify the intestinal microbiota. The type of milk used, the concentration of grains and milk, and whether an industrial or traditional fermentation process is utilized can all affect these changes (135, 136). In rats given a high-fat diet, kefir is said to lower the rate of *Firmicutes* and *Bacteroidete*s in feces, which in turn lowers abdominal fat mass, blood triglyceride levels, and lipoprotein lipase gene expression in adipose tissue. Consequently, it is believed that kefir’s modification of the composition of the gut microbiota will alter metabolic markers and lipogenesis (137). The degree of necrotic degeneration and macrophage infiltration in the intestine was also found to decrease as this ratio did. Thus, it has been shown that kefir can relieve inflammation in the intestine. By maintaining intestinal barrier integrity and inhibiting ileal inflammatory chemicals, kefir may prevent to mucosal leakage and systemic inflammation (133). The health effects and possible mechanisms of kefir are shown in Table 2.

**Table 2:** Kefir metabolites and their possible effects on health.

Possible health effects	Bioactive compounds	Mechanism of action
Anti-carcinogenic (108)	Kefiran EPS Lactic and acetic acids Bioactive peptides Extracellular vesicles	Using apoptosis to stop tumor growth, immune response, gut microbiota modification, Decreased DNA damage and tumor development The process of antioxidation
Anti-inflammatory (133, 198)	Kefiran EPS Extracellular vesicles Lactic acid Acetic acid Polysaccharide extract Pyruvic acids	Reduction of pro-inflammatory and molecular markers Reduction inflammatory infiltrate Modulation of gut microbiota Humoral and cellular immunity activation Controlling oxidative stress
Antioxidant (199)	EPS (Kefiran) Bioactive peptides Phenolic compounds	Clearing free radicals Reduction of ferric ion
Modulation of gut microbiota (137)	EPS (Kefiran) Bioactive peptides	Microorganism interactions Reducing the rate of Firmicutes and Bacteroidetes
Affecting the nervous system (47, 200)	EPS Bioactive peptides	Decreased depressive-like behavior by causing an increase in brain brain-derived neurotrophic factor levels Pathways involving bioactive peptides
Reducing blood pressure (201)	Bioactive peptides	Altering the ACE structure to stop angiotensin I from becoming angiotensin II
Osteoporosis (202)	Bioactive peptides	Reducing oxidative stress and inflammatory responses
Anti-diabetic (133, 203)	Bioactive peptides	Inhibitory activity of the α-glucosidase enzyme Healing effect on the insulin signaling pathway of the liver.
Affect blood lipids (133, 137, 204)	With LAB activity	Reducing the absorbability of cholesterol by changing its structure By affecting hepatic lipogenesis Reducing the rate of *Firmicutes* and *Bacteroidetes* in feces
Antibacterial (111, 203, 205)	Organic acids (Lactic acid) EPS (Kefiran) Bioactive peptides S-layer proteins Biofilm	Inhibiting bacterial cell metabolism by increasing pH Affecting cell membrane permeability
Anti-fungal (206)	EPS (Kefiran)	Decreasing the fungus’s capacity to stick to the intestinal barrier

The type of kefir, the conditions under which it is produced, and the amount of microorganisms present can all affect the bioactive components (lipidomics and proteomics) on which it exhibits its advantageous effects. For instance, proteomics derived from *κ*-, αs1-, and αs2-caseins were found in a kefir made from cow’s milk that primarily contained *Lactobacillus* spp., *Lactococcus* spp., *Leuconostoc* spp., *Saccharomyces* spp., and *Acetobacter* spp. These proteomics could potentially be beneficial to human health (138). In a study examining the proteomics of kefirs made from goat milk and fermented for 12, 24, and 36 h, 2,328 peptides and 11 bioactive peptides were identified. These peptides were assigned to β-casein in four cases, κ-casein in three, αs2-casein in two, αs1-casein in one, and β-lactoglobulin in the other. Of the 11 peptides, five were shown to have inhibitory effect against the angiotensin-converted enzyme (ACE), three to have antimicrobial activity, one to be antioxidative, another to be antithrombotic, and the final one to be an inhibitor of dipeptidyl peptidase IV. Nonetheless, it has been reported that the length of fermentation affects the quantities of these peptides. There are two bioactive peptides in kefir that has been fermented for 12 h, 10 in kefir that has been fermented for 24 h, and seven in kefir that has been fermented for 36 h, according to research (139). About 300 lipid types were found in a study that looked at the lipid composition of commercial kefirs. The following is a list of some of these: phosphatidylcholines (PCs), lyso-phosphatidylcholines (LPCs), phosphatidylethanolamines (PEs) andlyso-phosphatidylethanolamines (LPEs), phosphatidylserines (PSs), phosphatidylglycerols (PGs), phosphatidylinositols (PIs), lizil-fosfatidilgliserollerin (LyPG’ler), C18:3, C18:2, C18:1 [∆9-cis (oleic acid) and ∆11-trans (vaccenic acid)], C18:0 (stearic acid), C16:0 (palmitic acid), and C14:0 (myristic acid) (140). In another study examining the metabolomics of kefirs fermented for 8, 24 and 36 h, 722, 760, and 767 metabolites were detected, respectively. Acetyl-CoA and pyruvate have been shown to play a significant role in the TCA cycle, one of the metabolic pathways that is effective during the synthesis of these metabolites. According to the research, the breakdown of arginine and proline to spermine, leucine and isoleucine to L-asparagine, tyrosine to acetoacetate, and tryptophan to indoleacetaldehyde are the primary pathways of amino acid metabolism. Pathways involved in lipid metabolism have been reported to mainly involve glycerophospholipid and sphingolipid degradation. It has been demonstrated that the synthesis of folate, tropane, piperidine and pyridine alkaloid, glucosinolate, and phenylpropanoid are pathways associated with the biosynthesis of secondary metabolites (120).

In addition to kefir, another important fermented beverage is koumiss. Known to have originated in the Asian region, koumiss is now widely consumed. It is known that kumiss prepared from mare’s milk has a rich bioactive component content. It is known that it generally consists of LAB and yeasts. As LAB, it contains *Lactobacillus delbrueckii* ssp. *bulgaricus*, *Lactobacillus casei*, *Streptococcus Lactis* subsp. *lactis*, *Lactobacillus lactis* ssp. *lactis*, *Lactobacillus leichmannii*, *Lactobacillus delbrueckii* ssp. *lactis*, *Lactic streptococci*, *Lactobacillus acidophilus*. The yeast content can be composed of *Saccharomyces lactis*, *Rhodotorula*, *Torula lactis*, and *Torula koumiss*. However, this content may vary depending on the region and environmental conditions where the milk is produced. The bioactive components of koumiss may also vary depending on the microorganisms it contains. But generally speaking, it is rich in amino acids such as proline, lysine, tyrosine, valine and leucine, minerals such as lactose, linoleic and linolenic acid, phosphorus and calcium, and vitamins such as C, A, B, B2, B12, E, and pantothenic acid. With this content, it is known to have anticarcinogenic, hypocholesterolemic, antioxidant and antibacterial properties (141, 142).

## 7 Cheese

In essence, cheese is a dairy product that is made when milk ferments. Although it varies depending on the cheese type, its production begins by pasteurizing the milk at an average of 65 °C, then it is cooled to approximately 30 °C for coagulation. Afterward, the starter culture is added and fermented. After fermentation, salt, and renin enzyme are added and left to ripen (143). All milk components—carbohydrates, proteins, and lipids—change as a result of microbial activity during the ripening and cheese making processes. Enzymes, microorganisms that are either naturally present in cow’s milk or added as starters, coagulation and ripening conditions, and the composition of the milk all influence the dynamic biochemical process that determines the flavor and aroma of cheese (144). In addition, it is known that environmental conditions such as pH, temperature, salting stage, and ripening temperature in cheese production also affect the microorganism metabolism and production of aroma compounds in cheese (145).

Through the metabolism of starting or non-starter cultures, endogenous enzymes, coagulation enzymes, accessory enzymes, and ripening accelerating chemicals, cheese undergoes a variety of biochemical and microbiological changes during the ripening process. There are three primary types of biochemical reactions that take place during the ripening of cheese: the breakdown of lactose and citrate into organic acids and other components (glycolysis); the breakdown of proteins into amino acids and other amine products (proteolysis); and the breakdown of fat into fatty acids and other lipolysis compounds. Depending on the type of cheese, different metabolites may be produced in the finished product as a result of the metabolic processes that take place in the cheese. However, free amino acids and organic acids like lactic acid, citric acid, propionic acid, and acetic acid generally increase in concentrations throughout the fermentation and ripening of cheese. In addition, while there is an increase in some fatty acids, such as myristic acid, stearic acid, palmitic acid, and oleic acid, the level of some fatty acids, such as lauric and linoleic acid, may not increase (143).

Microorganisms, naturally found in the milk and starter cultures, constitute the microorganism content of the cheese. LAB, naturally found in the milk generally used in cheese production, constitute the most essential microorganism group in the cheese content. Non-starter LAB in cheese are generally facultative heterofermentative lactobacilli (144).

This content is also influenced by the microorganisms that is employed as a starter in regular cheese making. LAB, which are present in raw milk, make up the majority of the microorganisms in cheese, however the quantity varies by kind. Among these, the *Lactococcus* and *Streptococcus* genera are generally more abundant than those such as *Lacticaseibacillus* and *Lactobacillus* (144). Microorganisms such as *Streptococcus thermophilus*, *Lactobacillus delbrueckii* subsp. *lactis, Lactobacillus helveticus*, *Streptococcus gallolyticus* subsp. *macedonicus, Limosilactobacillus fermentum*, *Lacticaseibacillus casei*, *Lacticaseibacillus paracasei*, *Lacticaseibacillus rhamnosus*, *Lactiplantibacillus plantarum* subsp. *plantarum*, *Propionibacterium freudenreichii*, *Penicillium roqueforti*, *Penicillium candidum* can generally be found in cheese (146). According to a study comparing the quality and microorganism content of Cheddar cheese, while *Thermus* (0.5%), *Pediococcus* (0.4%), and *Pseudomonas* (0.007%) were found to be low, *Streptococcus* (53%), *Lactobacillus* (30%), and *Lactococcus* (15%) were dominant in good quality cheeses. In lower-quality cheeses, *Lactococcus* (81%) was dominant, while *Lactobacillus* (15%) and *Streptococcus* (2%) were found to be lower (147). Semi-solid cheese typically has 54% moisture, 48% fat, and 42% protein, though the exact amounts vary based on the variety and production conditions (145).

Cheese undergoes numerous physical and chemical changes as it ferments and ripens. The primary source of these changes is the starter and non-starter microorganisms in the cheese. Various metabolic events caused by these microorganisms form different types and amounts of metabolites throughout the process. While these metabolites give cheese flavor, they are also compounds that can affect health (148).

Lipids and nitrogenous components are essential for the development of cheese flavor, regardless of the type of cheese. Natural enzymes from the milk used in production, milk coagulants, and starter or non-starter microorganisms all catalyze the proteolysis of proteins. Initiator or non-initiator enzymes are crucial for the production of short peptides and amino acids that are the building blocks of the taste compounds found in cheese, whereas coagulant enzymes are primarily in charge of hydrolyzing caseins into big or intermediate peptides. Lactate catabolism facilitates glycolysis, but citrate catabolism is mostly finished in the first or second week of ripening. Additionally, during ripening, free fatty acid catabolism and lipolysis take place. Different levels of peptides, amino acids, free fatty acids, and volatile substances are released in cheeses as a result of these three crucial biological events (149). The amount or generation of these chemicals is also influenced by the type of cheese and the microorganisms it contains. For instance, soft-type matured cheeses containing *Aspergillus oryzae* and *Aspergillus sojae* were shown to have higher levels of lactic acid, amino acids, and acetoin and lower levels of methyl ketones and volatile fatty acids (150).

The primary carbon sources of the starter microorganisms are lactose and citric acid during cheese fermentation. Beta-galactosidase breaks down lactose into galactose and glucose. While glucose fuels glycolysis, galactose and citric acid metabolisms occur in a strain-dependent manner. Prior to glycolysis, galactose is transformed into glucose-6-phosphate. Pyruvate is produced from citric acid. The acetolactate resulting from the conversion of pyruvate produces butanediol and diacetyl, which create a buttery taste (151).

One of the most critical steps in cheese production is lactic acid formation. All varieties of cheese require the conversion of lactose to lactic acid. Lactic acid causes pH to decrease and the development of an acidic taste. LAB are crucial for the glycolysis-based fermentation process, which converts lactose to lactate. Then, lactate is converted to acetate and CO_2_. Most of this conversion occurs in the early stages of fermentation. The principal organic acid found in cheeses, lactic acid, serves the primary purpose of inhibiting the growth of undesired microorganisms, particularly in their early stages. Non-starter LAB transform lactic acid in cheese into DL-lactate, or in the presence of *Propionibacterium* spp., pro-pionate, acetate, H_2_O, and CO_2_. *Penicillium* species and yeasts may simultaneously metabolize this lactic acid to produce H_2_O and CO_2_, while non-starter LAB can produce formate, acetate, and CO_2_. Lactic acid can also be metabolized to butyrate, H_2_, and CO_2_ by *Clostridium* spp. (149). Apart from LAB, *Propionibacterium freudenreichiiis*, used in cheese, can convert lactic acid into propanoic acid, acetic acid, and carbon dioxide. Meanwhile, the conversion of citrate to acetic acid is also active. *Enterococcus faecium*, a starter, is primarily responsible for converting citric acid to diacetyl, and excessive diacetyl production early in the cheese-making process influences the development of butter flavor (152). After 4 weeks of ripening, the largest amount of diacetyl is produced. However, as ripening progresses, the amount of diacetyl drops because LAB convert it to acetoin. Citrate metabolism results in the appearance of diacetyl, acetoin, acetate, 2,3-butanediol, and CO_2_. (145).

Rennet is the primary beginning point for the proteolysis process, which starts during the production stage of cheesemaking. Primary and secondary proteolysis are the two methods used to study proteolysis. The breakdown of casein by chymosin or milk-based enzymes such plasmin and cathepsin D is known as primary proteolysis. Using protease and peptidase enzymes from starting and non-starter bacteria, secondary proteolysis hydrolyzes the byproducts of primary proteolysis into smaller peptides and amino acids (149). Proteinases, peptidases, milk plasmin, and the coagulant all work together to produce free amino acids and short peptides (153). Amino acids and small peptides give cheeses their distinctive flavor. Numerous soluble and volatile chemicals are also produced by catabolizing free amino acids. These peptides also exhibit antioxidant, antihypertensive, mineral-binding, immunomodulatory, and antibacterial qualities (154).

The metabolic activities of microorganisms in cheese may also affect the formation of some biogenic amines. For example, Microbial glutamate decarboxylase can cause the synthesis of GABA, a biogenic amine, and tyrosine decarboxylase can cause the conversion of tyrosine to tyramine. These enzymes might also be involved in the synthesis of beta-phenylethylamine. Additionally, enzymes such as arginine decarboxylase, agmatine deaminase, N-carbamoylputrescine amidase, and ornithine decarboxylase may affect the production of putrescine, another biogenic amine (155).

Another crucial component for the development of cheese flavor during ripening is milk fat. Free fatty acids are produced when lipolytic esterases hydrolyze milk fat. Fatty acids contribute to developing a sharp and sour aroma in cheese taste. Additionally, they function as precursors to several fragrance chemicals, including lactones, esters, secondary alcohols, and methylketones. The main source of the lipolytic enzymes in cheese is microorganisms (145). High concentrations of volatile fatty acids can make the cheese more acidic overall. As a result, cheese’s level of protective microflora rises in tandem with its free fatty acid content. In addition, short-chain fatty acids especially contribute greatly to the formation of the aroma of cheeses (149).

Numerous metabolites produced during the cheese-making process are crucial in preventing disease. It is also well recognized that cheese’s microbes play a crucial role in maintaining health. Human health benefits from the usual microbiota found in cheeses include immune system regulation, gut-brain barrier strengthening, reduced carcinogenic effects, antibacterial activity against harmful microorganisms, and cholesterol-lowering effects (156). Metabolites in different cheese varieties detected in some studies are shown in Table 3.

**Table 3:** Metabolites contained in various types of cheese in the literature.

Cheese	Microorganism content	Metabolites
Lighvan cheese (207) A semi-hard cheese Natural lamb rennet or commercial microbial rennet were added to raw milk at 22–28 °C. Ripening take place in 4 months under temperature (8–12 °C) and humidity (80–85%) conditions.	Aerobic mesophiles microbiota *Enterobacteriaceae* *Lactococci* *Lactobacilli* *Enterococci*	Lactic acid, Caproic acid, Valine, Urea, Valeric acid Glycerol, Isoleucine, GABA, Arabitol, Putrescine, Cadaverine, Myo-Inositol, Lysine, Stearic acid, Palmitic acid, Melibiose, Succinic acid, Threonine, Capric acid, Glutamic acid, Tryptamine Methionine, Alanine, Phenylalanine, Tyramine, Ornithine, Galactose, Dopamine, Tyrosine, Oleamide, Lactose
Monascus-ripened cheese (208) Yeast and *Monascus fumeus* were added to pasteurized milk with a fat content of 3.2 g/L. The cheese was incubated for 37 days at 10 °C after being stored for 5 days at 26 °C and 90% relative humidity.	Lactic acid bakteria *Monascus fumeus Ligilactobacillus salivarius*	organic acids, lipids, fatty acyls, sterol lipids, carboxylic acids, prenol lipids, glycerophospholipids, 20-hydroxyeicosatetraenoic acid, 20-HETE ethanolamide, 3-hydroxy-N6, N6-trimethyl-L-lysine, uridine diphosphate-N-acetylglucosamine, 20-carboxy-leukotriene, 3B,12a-dihydroxy-5a-cholanoic acid, 25-hydroxyvitamin D2-25-glucuronide, tomatidine, lichesterol, valine, Ser-Ile-OH, lysyl-glycine, 2-methylbutyroylcarnitine, butyryl-L-carnitine, 4-oxo-13-cis-retinoate, N-phenylacetylaspartic acid
Beaten cheese (209) After raw cow milk containing 3.9% fat was pasteurized, starter culture were added and the cheese was ripened for 180 days.	*Streptococcus thermophilus* *Lactobacillus delbrueckii* subsp. *bulgaricus* *Lacticaseibacillus casei* *Lactococcus lactis* subsp. *lactis* *Lactococcus lactis* subsp. *cremoris*	Acetic acid, Butanoic acid, Hexanoic acid, Octanoic acid, Ethanol, 2-Butanol, 3-Methyl-1-butanol, 1-Hexanol, 2-Heptanol, Ethyl acetate, Ethyl butanoate, 3-Methyl-1-butylacetate, Ethyl hexanoate, Ethyl octanoate, 2-Propanone, 2-Butanone, 3-Hydroxy-2-butanone, 3-Methylbutanal, a-Pinene, Toluene, Limonene
Turkish white cheese (143) The culture was introduced to pasteurized full-fat cow’s milk and allowed to mature for 100 days.	*Lactococcus lactis* subsp. *lactis Lactococcus* *lactis* subsp. *cremoris*	Lactic, Citric, Propionic, Acetic, Lauric acid, Myristic acid, Palmitic acid, Stearic acid, Oleic acid, Linoleic acid, Glycine, Alanine, Valine, Isoleucine, Methionine, Proline Phenylalanine, Tyrosine, Tryptophan, Serine, Threonine, Cysteine, Lysine, Histidine, Aspartic acid Glutamic acid, Asparagine, Glutamine
Cheddar (147) The cheese takes a full year to mature.	*Streptococcus Lactobacillus Lactococcus* *Thermus* *Pediococcus* *Pseudomonas*	Alanine, Glycine, Serine, Threonine, Piperidine, GABA, Pipecolic acid, Ornithine, Asparagine, Glutamine, Histidine, Tryptophan, Valine, Leucine, Isoleucine, Proline, Aspartic acid, Methionine, Pyroglutamic acid, Cysteine, Glutamic acid, Phenylalanine, Lysine, Tyrosine, Myristic acid, Stearic acid, Palmitic acid, Octadecanol, Succinic acid, Glyceric acid, Lactic acid, Glycerine-3-phosphate, Inositol myo, Sucrose, Lactose.
Swiss cheese (159) The cheese was left to mature for 9 months	*Propionibacterium freudenreichii*	Lactic acid, Butyric acid, Glucose, Propionic acid, Oleic acid, palmitic acid, miristik acid, acetoin, acetic acid, 2-heptanone, 2-pentanone, tetramethylpyrazine, D-Limonene, 2-Methyl-1-butanol, benzaldehyde, methylethyl acetic acid
Mold ripened cheese The effect of using Penicillium species as starter culture was investigated (162) Mold ripening cheeses sold in supermarkets were examined (164)	*Penicillium camemberti, Penicillium roqueforti* ve *Geotrichum candidu* *Dipodascus, Penicillium, Debaryomyces, Kluyveromyces, Candida, Actinobacteria, Firmicutes, Proteobacteria*	Lactic acid, formic acid, acetic acid, succinic acid, butanoic acid, 3-Methyl-butanoic acid, hexanoic acid, octanoic acid, malic asit Ethanol, 3-Methyl-1-butanol, 2,3-Butanediol, phenol, geosmin, n-Heptanol, pentanol, n-octane, styrene, n-Nonly acetate, heptyl acetate, o-xylene, 2-Methylnaphthalene, 1-Methylnaphthalene, hexadecane 3-Octanone, methenamine, 3-Methylpyridine, nonanal, acetoin, 2-Pentanone, 2-Heptanon
Parmesan (167) The cheeses examined were matured for 12, 24 and 36 months	*Lactobacillus delbrueckii*, *Lactobacillus helveticus, Lactobacillus paracasei*, and *Streptococcus thermophilus*	Acetic acid, benzeneacetaldehyde, heptanoic acid, L-Lactic acid, n-Decanoic acid, nonanal, octanoic acid, trisiloxane 1,1,3, butanoic acid, acetone caprylic acid, lauric acid, palmitic acid, stearic acid, linoleic acid, 7-Acetylintermedine, Asparaginyl-Tryptophan, Asparaginyl-Tryptophan, Asparaginyl-Tryptophan, Serylmethionine, N-Methyl-D-aspartic acid, fabatin, 2-(1-Naphthyl)acetamide, N-palmitoyl alanine, 1-Ethylhexyl tiglate, Dodecanamide, S-adenosyl-L-methionine, Orotidylic acid, N1-Acetylspermidine, Dehydrospermidine, junosine, Prostaglandin E-2, N-Acetylputrescin
Cottage cheese (169) Milk is fermented at 34 °C for cheese making	*Lactococcus lactis* subsp. l*actis, L. lactis* subsp. *cremoris*	Acetic acid, butyric acid, hexanoic acid, octanoic acid, ethanol, 1-Hexadecanol, benzaldehyde, acetoin, ethyl acetate, toluene, proline, phenylalanin

### 7.1 Swiss cheese

Swiss cheeses are cooked and ripened cheeses that undergo propionic acid fermentation, usually achieved by adding a culture of strains of *Propionibacterium freudenreichii*. A traditional Swiss cheese is made using cow’s milk. This cheese, which is widely consumed all over the world, is matured for a long time (at least 60 days, up to 9 months) and has a hard structure (157). In Swiss cheeses, especially *Propionibacterium freudenreichii* strains are important starter cultures (158). Factors such as the type and quality of milk used during the production of Swiss cheese, the diversity of the starter culture, and the geography in which it is produced greatly affect the metabolite content and amount in the final product. Generally, a classic Swiss cheese contains high amounts of organic acids such as lactic acid, butyric acid, glucose, and propionic acid. Additionally, oleic acid, palmitic acid, and myristic acid contents can also be seen to be high. In addition, a classic Swiss cheese was also found to be rich in acetoin, acetic acid, 2-heptanone, 2-pentanone, tetramethylpyrazine, D-Limonene, 2-Methyl-1-butanol, benzaldehyde, methylethyl acetic acid (159).

### 7.2 Mold ripened cheeses

Mold ripened cheeses are usually white and soft cheeses. The main microflora covering the cheese surface is *Penicillium camemberti*. *Penicillium roqueforti* and *Geotrichum candidum* are other cultures used in production (160). Mold ripened cheeses are generally divided into two categories: surface mold-ripened cheeses (or bloomy rind) or blue-veined cheeses. Blue-veined cheese is characterized by the presence of bluish-green mold (*Penicillium roqueforti*) all over and is matured for at least 60 days (161). While the organic acid content of these cheeses is generally the highest in lactic acid, it is also known that they contain formic acid, acetic acid, succinic acid, butanoic acid, 3-Methyl-butanoic acid, hexanoic acid, octanoic acid, and malic acid (160). Additionally, metabolites such as ethanol, 3-Methyl-1-butanol, 2,3-Butanediol, phenol, geosmin, n-Heptanol, pentanol, n-octane, styrene, n-Nonly acetate, heptyl acetate, o-xylene, 2-Methylnaphthalene, 1-Methylnaphthalene, hexadecane may be present. In addition to this, 3-Octanone, methenamine, 3-Methylpyridine, nonanal, acetoin, 2-Pentanone, 2-Heptanone may be observed depending on the type of starter culture used (162–164).

### 7.3 Parmesan

Parmesan is a type of cheese that uses a starter culture mixture, usually consisting of thermophilic bacteria, and to which lipases are often added to enhance the development of its distinctive aroma. This hard cheese, with a low moisture content, is matured for at least 9 months (it can be matured for up to 2 years). *Lactobacillus delbrueckii*, *Lactobacillus helveticus, Lactobacillus paracasei*, and *Streptococcus thermophilus* are the microorganisms used in the production of Parmesan (165). Metabolites such as acetic acid, benzeneacetaldehyd, heptanoic acid, L-Lactic acid, n-Decanoic acid, nonanal, octanoic acid, trisiloxane 1,1,3, butanoic acid, acetone may be present in different amounts in Parmesan cheese depending on its ripening time. In addition, fatty acids such as caprylic acid, lauric acid, palmitic acid, stearic acid, and linoleic acid can also be found (166). 7-Acetylintermedine, Asparaginyl-Tryptophan, Asparaginyl-Tryptophan, Asparaginyl-Tryptophan, Serylmethionine, N-Methyl-D-aspartic acid, fabatin, 2-(1-Naphthyl) acetamide, N-palmitoyl alanine, 1-Ethylhexyl tiglate, Dodecanamide, S-adenosyl-L-methionine, Orotidylic acid, N1-Acetylspermidine, Dehydrospermidine, junosine, Prostaglandin E-2, N-Acetylputrescine are another compounds that can be found in Parmesan (167).

### 7.4 Cottage cheese

Cottage cheese, considered a fresh cheese, is a soft cheese type that generally has a low energy content. Long maturation periods are not common in its production. In the production of cottage cheese, cultures that ferment lactic acid and citric acid are often used together. Traditionally produced using *Lactococcus lactis* subsp. *lactis* ve *L. lactis* subsp. *cremoris* strains (168). Depending on the type of strain used in cottage cheese, different metabolites may occur. These metabolites may include acetic acid, butyric acid, hexanoic acid, octanoic acid, ethanol, 1-Hexadecanol, benzaldehyde, acetoin, ethyl acetate, toluene, proline, phenylalanine (169).

## 8 Lactose hydrolyzed dairy products

Novel fermented dairy products have emerged as a global availability recently. In addition to the positive health effects of fermented dairy products on their own, these products have health effects due to processes such as changing these products in various ways, enriching them, or adding substances with an antioxidant profile (170). In this context, firstly, lactose-free fermented dairy products can be considered. Lactose is a disaccharide composed of galactose and glucose, and its molecular structure is described as O-ß-D-galactopyranosyl-(1–4)-ß-D-glucose. The production of lactose occurs exclusively in the mammary gland, and it involves the transfer of a galactose molecule coupled to UDP to a glucose molecule, facilitated by a galactosyl transferase enzyme (171). A common sugar in most mammals’ milk is lactose, which makes up around 5% of bovine milk, and 7% of human milk (172). Lactose intolerance is a genetic condition characterized by a reduced ability to digest and absorb lactose, a sugar in dairy products. A decrease in the lactase enzyme, which occurs after weaning, and damage to the lining of the digestive tract can be responsible for lactose intolerance (173). The primary approach to managing lactose intolerance in individuals is to substitute normal dairy products with low-lactose and lactose-free alternatives; dairy products supplemented with exogenous lactase or probiotics (174). Consumption of low-lactose or lactose-free dairy products can decrease gastrointestinal symptoms in those with lactose intolerance, while still providing the essential nutrients in milk (175). As lactose intolerance becomes more common and people become more aware of it, there is a growing demand for lactose-free or low-lactose products (176).

Currently, generally used methods for decreasing lactose content are enzymatic hydrolysis of lactose, membrane filtering, and fermentation. Lactose-free milk can undergo further processing to produce lactose-free or low-lactose yogurt, cheese, milk powder, ice cream, and other dairy products (177). Generally, two methods (batch and aseptic) are used to manufacture lactose-free milk, and both methods utilize soluble lactase enzymes (178). The conventional choice for the manufacturing of lactose-free dairy products has been the neutral β-galactosidase enzyme obtained from the dairy yeast *Kluyveromyces lactis* (*and its close relatives Saccharomyces lactis, K. marxianus or K. fragilis*) (179).

Enzymatic hydrolysis can affect the fermentation process and the final dairy products’ functional qualities in addition to just eliminating lactose. Therefore, lactose hydrolyzation may affect the properties of fermented dairy products. The overall presence of *Lactobacillus delbrueckii* subsp. *bulgaricus* 2038, the level of EPS, and the viscosity in the co-culture of *Lactobacillus delbrueckii* subsp. *bulgaricus* 2038 and *Streptococcus thermophilus* 1,131 were significantly higher in lactose-hydrolyzed milk compared to unhydrolyzed milk (180).

By increasing the growth and metabolic activity of particular bacterial cultures, the use of lactose-hydrolyzed milk has the potential to enhance the textural attributes of these products and potentially amplify their health advantages. As a result, lactose-hydrolyzed dairy products are an exceptional option for individuals who are lactose-intolerant or who are in search of nutritionally enriched food alternatives.

## 9 Probiotic/prebiotic fortified dairy products

Fermented dairy products are fortified with components like probiotics and prebiotics. Although certain fermented dairy products may contain naturally occurring probiotics, ‘probiotics’ and ‘fermented food’ are not synonymous, and the term ‘probiotic’ should be exclusively used when there is a proven health advantage provided by thoroughly identified and described living microorganisms (181). Dairy products enhanced with probiotics are becoming more and more popular because of their health advantages. Aljutaily et al. (182) looked into how body weight and gut microbiota were affected by Greek-style yogurt and cottage cheese that contained probiotics made from cow or goat milk. In comparison to the cow cheese groups, probiotic goat yogurt increased weight at day 28, while probiotic cow cheese decreased weight gain at days 28 and 35. Order *Clostridiales*, family *Lachnospiraceae*, genus SMB53, and species *Ruminococcus gnavus* were all more prevalent in the probiotic cow yogurt group (182). Because these organisms are known to produce butyrate, they may be able to prevent metabolic diseases and obesity (183). Conversely, the probiotic cow yogurt group showed decreased levels of family *Erysipelotrichaceae, Clostridiaceae*, genus *Oscillospira*, *Clostridium, Ruminococcus, Aerococcus*, species *Ruminococcus gnavus* and *Clostridium perfringens* compared to the regular cow yogurt group. In probiotic cow cheese, the relative abundance of order *Clostridiales*, family *Ruminococcaceae*, S24-7, *Lachnospiraceae* and species *R. gnavus* increased significantly versus regular cow cheese. The probiotic cow cheese group showed the greatest enhancement, with 30 OTUs, suggesting that probiotic cow cheese may be an effective vehicle for probiotic delivery (182).

Fermented dairy products can be enriched with different prebiotics and bioactive ingredients to improve gut health and overall functionality (184–194). Three fundamental components comprise the concept of the prebiotics: a substance, a physiologically beneficial effect, and a mechanism mediated by microbiota (185). Milk fermented with *Lactobacillus helveticus* ASCC 511 (LH511) and enriched with citrulline improved the function of the intestinal epithelial barrier and reduced inflammation in IPEC-J2 cells produced by pathogenic *Escherichia coli*. The resistance across the epithelial layer (TEER) and expression and distributions of the proteins involved in tight junctions (ZO-1, occludin, and claudin-1), receptors responsible for detecting pathogens (TLR2 and TLR4), and molecules that inhibit the TLR signaling pathway increased (A20 and IRAK-M) (186). In animal models, goat milk yogurt supplemented with red rice bran flour was tested, and goat milk fermentation generated bioactive peptides with antioxidant, antibacterial, and antihypertensive properties. While, rice bran is antioxidant-rich and fiber-rich; potential antioxidant components in red bran rice include phenolic acid, flavonoids, anthocyanins, proanthocyanin, vitamin E, *γ*-oryzanol, and folic acid. As a result, Serum Glutamate Pyruvate Transaminase (SGPT) was reduced by goat milk yogurt with red rice bran flour in addition to lowering creatinine levels and promoting renal health (187, 188). Adding inulin to skim milk, even at a low concentration, greatly enhances the growth and survival of *Lactobacillus acidophilus*, *Lacticaseibacillus rhamnosus*, and *Bifidobacterium lactis* in non-fat fermented milk (189). Moreover, the addition of inulin as a prebiotic to yoghurt enhanced the functional properties and the viability of *Bifidobacterium bifidum* (184). Seed mucilage possesses prebiotic characteristics enhances the proliferation of LAB and promotes advantageous gut microbiota while suppressing pathogenic microorganisms (190, 191). Chia mucilage comprises planteose, a galactosyl sucrose oligosaccharide that exhibits prebiotic properties, fostering advantageous gut microorganisms. Chia seed mucilage is regarded as an innovative functional ingredient for the food industry (192, 193). In a study of Hovjecki et al. (194), cow milk yoghurt with different concentrations (1, 5 and 3%) chia seeds mucilage were produced. In the yoghurt with the 3% concentration of chia mucilage *L. bulgaricus* increased. At the end of the storage period, the total amount of lactobacilli significantly increased compared to the control (194).

In addition to prebiotics, various metabolites can also enhance the functional properties of fermented dairy products. An *in vitro* study showed that kefir enhanced with aronia berry, significantly inhibited α-glucosidase activity, but had a lower impact on pancreatic α-amylase (195). This suggests that combining polyphenol-rich ingredients with fermented dairy products could offer additional health benefits. Propolis had a noticeable inhibitory impact on *Bifidobacterium animalis* subsp. *lactis* in probiotic yogurt; moreover, probiotic yogurts that contained 0.03% propolis and 2.5% cinnamon had a greater concentration of *Bifidobacterium animalis* ssp. *lactis* compared to other probiotic yogurts (cinnamon concentration of 0.3 and 1%). All probiotic yogurts containing a combination of propolis alone or in combination with cinnamon had a notable impact on the colony counts of *Lactobacillus acidophilus*. Propolis exhibited a substantial reduction in the quantity of *Lactobacillus delbrueckii* subsp. *bulgaricus* (196).

The effects of various substances vary based on the products’ microorganism profiles, matrix, and ingredients. Existing evidence generally shows that the effects of novel fermented dairy products are through gut modulation and metabolomics. However, due to the relatively new nature of the subject, human studies are limited in the literature, and further studies are required to elucidate the findings.

## 10 Conclusion

Fermented dairy products serve as an important source of health-promoting multibiotics such as probiotics, prebiotics, and postbiotics according to the accumulated literature. Probiotics improve the gut microbiota composition, regulate the immune system and synthesis of several bioactive metabolites. For prebiotics including GOS of fermented dairy products significantly vary according to plenty of factors such as the milk types and composition, enhance effects of the probiotics. Postbiotics including SCFAs, organic acids, bioactive peptides, and GABA show neuroprotective, immunomodulatory, anti-inflammatory effects. Besides, their composition and health effects are shaped by the interplay of milk source, fermentation processes, the addition of probiotics or functional ingredients, and whether a conventional or industrial fermentation procedure is employed. The many metabolites created throughout the fermented dairy-making process are promising for disease prevention. Advances in metabolomics and analytical methodologies are improving our understanding of the interactions within fermented milk and the role of them in personalized nutrition and preventive health; however, significant research gaps remain, particularly in the areas of lipidomics and proteomics. With their ability to control the immune system, fortify the gut-brain barrier, their antioxidant effects, etc., it is well recognized that the bioactive metabolites in fermented dairy foods play a significant role in preserving health via certain mechanisms of action.

To sum up, fermented dairy foods are one of the most popular food groups of recent times in terms of both food technology and health. The multibiotics and metabolites included in these foods without a doubt contribute significantly to this. These effects of fermented dairy products vary greatly depending on a number of parameters, including the kind and composition of the milk and the product. Ongoing investigation into their metabolomic changes will help clarify its potential in supporting metabolic health and inform future dietary guidelines.

## References

[1] Fermented Foods and Beverages Market Outlook (2022 to 2032). Available online at: https://www.futuremarketinsights.com/reports/fermented-foods-and-beverages-market (Accessed September 18, 2025).

[2] Ibrahim SA Yeboah PJ Ayivi RD Eddin AS Wijemanna ND Paidari S . A review and comparative perspective on health benefits of probiotic and fermented foods. Int J Food Sci Technol. (2023) 58:4948–64. doi: 10.1111/ijfs.16619

[3] Sarkar S Sha SP Ghatani K. Metabolomics of ethnic fermented foods and beverages: understanding new aspects through Omic techniques. Front Sustain Food Syst. (2023) 7:1040567. doi: 10.3389/fsufs.2023.1040567

[4] Hill C Tancredi DJ Cifelli CJ Slavin JL Gahche J Marco ML . Positive health outcomes associated with live microbe intake from foods, including fermented foods, assessed using the NHANES database. J Nutr. (2023) 153:1143–9. doi: 10.1016/j.tjnut.2023.02.019, PMID: 36822397 PMC10196563

[5] Sessou P Keisam S Gagara M Komagbe G Farougou S Mahillon J . Comparative analyses of the bacterial communities present in the spontaneously fermented milk products of Northeast India and West Africa. Front Microbiol. (2023) 14:1166518. doi: 10.3389/fmicb.2023.1166518, PMID: 37886068 PMC10598763

[6] Hadjimbei E Botsaris G Chrysostomou S. Beneficial effects of yoghurts and probiotic fermented milks and their functional food potential. Foods. (2022) 11:2691. doi: 10.3390/foods11172691, PMID: 36076876 PMC9455928

[7] Sakandar HA Zhang H. Trends in probiotic(s)-fermented milks and their in vivo functionality: a review. Trends Food Sci Technol. (2021) 110:55–65. doi: 10.1016/j.tifs.2021.01.054

[8] Savaiano DA Hutkins RW. Yogurt, cultured fermented milk, and health: a systematic review. Nutr Rev. (2021) 79:599–614. doi: 10.1093/nutrit/nuaa013, PMID: 32447398 PMC8579104

[9] Sun Y Guo S Wu T Zhang J Kwok LY Sun Z . Untargeted mass spectrometry-based metabolomics approach unveils biochemical changes in compound probiotic fermented milk during fermentation. NPJ Sci Food. (2023) 7:21. doi: 10.1038/s41538-023-00197-z, PMID: 37225736 PMC10209183

[10] Vinderola G Cotter PD Freitas M Gueimonde M Holscher HD Ruas-Madiedo P . Fermented foods: a perspective on their role in delivering biotics. Front Microbiol. (2023) 14:1196239. doi: 10.3389/fmicb.2023.1196239, PMID: 37250040 PMC10213265

[11] Shangpliang HNJ Tamang JP. Genome analysis of potential probiotic Levilactobacillus brevis AcCh91 isolated from Indian home-Made fermented Milk product (chhurpi). Probiot Antimicrob Prot. (2023) 16:1583–1607. doi: 10.1007/s12602-023-10125-y 37466831

[12] de Melo Pereira GV Carvalho Neto DP Maske BL De Dea Lindner J Vale AS Favero GR . An updated review on bacterial community composition of traditional fermented milk products: what next-generation sequencing has revealed so far? Crit Rev Food Sci Nutr. (2022) 62:1870–89. doi: 10.1080/10408398.2020.1848787, PMID: 33207956

[13] Okoniewski A Dobrzyńska M Kusyk P Dziedzic K Przysławski J Drzymała-Czyż S. The role of fermented dairy products on gut microbiota composition. Fermentation. (2023) 9:231. doi: 10.3390/fermentation9030231

[14] García-Burgos M Moreno-Fernández J Alférez MJM Díaz-Castro J López-Aliaga I. New perspectives in fermented dairy products and their health relevance. J Funct Foods. (2020) 72:4059. doi: 10.1016/j.jff.2020.104059

[15] Rosa MC Carmo MRS Balthazar CF Guimarães JT Esmerino EA Freitas MQ . Dairy products with prebiotics: an overview of the health benefits, technological and sensory properties. Int Dairy J. (2021) 117:5009. doi: 10.1016/j.idairyj.2021.105009

[16] Zoumpopoulou G Pot B Tsakalidou E Papadimitriou K. Dairy probiotics: beyond the role of promoting gut and immune health. Int Dairy J. (2017) 67:46–60. doi: 10.1016/j.idairyj.2016.09.010

[17] Srinivash M Krishnamoorthi R Mahalingam PU Malaikozhundan B Keerthivasan M. Probiotic potential of exopolysaccharide producing lactic acid bacteria isolated from homemade fermented food products. Journal of agriculture and food. Research. (2023) 11:1–15. doi: 10.1016/j.jafr.2023.100517

[18] Sharma H Ozogul F Bartkiene E Rocha JM. Impact of lactic acid bacteria and their metabolites on the techno-functional properties and health benefits of fermented dairy products. Crit Rev Food Sci Nutr. (2023) 63:4819–41. doi: 10.1080/10408398.2021.2007844, PMID: 34845955

[19] Suh JH. Critical review: metabolomics in dairy science-evaluation of milk and milk product quality. Food Res Int. (2022) 154:110984. doi: 10.1016/j.foodres.2022.110984, PMID: 35337558

[20] Zhang T Geng S Cheng T Mao K Chitrakar B Gao J . From the past to the future: fermented milks and their health effects against human diseases. Food Front. (2023) 4:1747–77. doi: 10.1002/fft2.304

[21] Khan MN Bashir S Imran M. Probiotic characterization of *Bacillus* species strains isolated from an artisanal fermented milk product dahi. Folia Microbiol (Praha). (2023) 68:757–69. doi: 10.1007/s12223-023-01048-w, PMID: 37055653

[22] Özbal H Breu A Thissen L Gerritsen F van den Bos E Galik A . From bowls to pots: the dairying revolution in Northwest Turkey, a view from Barcın Höyük, 6600 to 6000 BCE. PLoS One. (2024) 19:e0302788. doi: 10.1371/journal.pone.0302788, PMID: 38722837 PMC11081328

[23] Halawa A. Influence of the traditional food culture of ancient Egypt on the transition of cuisine and food culture of contemporary Egypt. J Ethnic Foods. (2023) 10:11. doi: 10.1186/s42779-023-00177-4

[24] Prajapati J Nair BM. The history of fermented foods In: Farnworth ER, editor. Handbook of fermented functional foods. Boca Raton, London, New York: CRC Press, Taylor & Francis Group (2008). 3–4.

[25] Widyastuti Y Febrisiantosa A Tidona F. Health-promoting properties of lactobacilli in fermented dairy products. Front Microbiol. (2021) 12:673890. doi: 10.3389/fmicb.2021.673890, PMID: 34093496 PMC8175972

[26] Abi Khalil R Yvon S Couderc C Jard G El Rammouz R Abi Nakhoul P . Traditional fermented milk products of eastern Mediterranean countries: a cultural heritage to preserve. Int Dairy J. (2023) 147:105768. doi: 10.1016/j.idairyj.2023.105768

[27] Kalam Saleena LA Phing PL Gan R-Y Al-Nabulsi A Osaili T Kamal-Eldin A . Fermented dairy products from middle eastern and northern African (MENA) countries: insight on production and physiochemical characteristics. Int Dairy J. (2023) 141:105614. doi: 10.1016/j.idairyj.2023.105614

[28] Narvhus JA Abrahamsen RK. Traditional and modern Nordic fermented milk products: a review. Int Dairy J. (2023) 142:1–15. doi: 10.1016/j.idairyj.2023.105641

[29] Pohsnem JM Ramakrishnan E Parasar DP. Fermented food products in the Himalayan belt (north East India) and their health benefits. International journal of gastronomy and food. Science. (2023):31. doi: 10.1016/j.ijgfs.2023.10067637797005

[30] Konuspayeva G Baubekova A Akhmetsadykova S Faye B. Traditional dairy fermented products in Central Asia. Int Dairy J. (2023) 137:5514. doi: 10.1016/j.idairyj.2022.105514

[31] Bintsis T Papademas P. The evolution of fermented milks, from artisanal to industrial products: a critical review. Fermentation. (2022) 8:679. doi: 10.3390/fermentation8120679

[32] Mallappa RH Balasubramaniam C Nataraj BH Ramesh C Kadyan S Pradhan D . Microbial diversity and functionality of traditional fermented milk products of India: current scenario and future perspectives. Int Dairy J. (2021) 114:104941. doi: 10.1016/j.idairyj.2020.104941

[33] Xia A Jiang Y Li B Wang T Zhao J Liu X . Indigenous Chinese fermented dairy products: microbial diversity, flavour, and health benefits. Int Dairy J. (2022) 135:5479. doi: 10.1016/j.idairyj.2022.105479

[34] Guo S Sun Y Wu T Kwok LY Sun Z Wang J . Co-fermented milk beverage has better stability and contains more health-promoting amino acid metabolites than single-strain-fermented milk beverage over one-month storage. Food Chem. (2024) 430:136840. doi: 10.1016/j.foodchem.2023.136840, PMID: 37541038

[35] Turkmen N Akal C Özer B. Probiotic dairy-based beverages: a review. J Funct Foods. (2019) 53:62–75. doi: 10.1016/j.jff.2018.12.004

[36] Paul S Hossain TJ Ali F Hossain ME Chowdhury T Faisal IK . Assessment of the in-vitro probiotic efficacy and safety of *Pediococcus pentosaceus* L1 and *Streptococcus thermophilus* L3 isolated from Laban, a popular fermented milk product. Arch Microbiol. (2024) 206:82. doi: 10.1007/s00203-023-03812-5, PMID: 38294545

[37] El-Menawy RK Mohamed DM Ismail MM Hassan AM. Optimal combination of cow and quinoa milk for manufacturing of functional fermented milk with high levels of antioxidant, essential amino acids and probiotics. Sci Rep. (2023) 13:20638. doi: 10.1038/s41598-023-47839-6, PMID: 38001129 PMC10673919

[38] Mangia NP Carta S Murgia MA Montanari L Nudda A. Fermented milk produced with goat milk enriched with PUFA omega-3 by supplementation of diet with extruded linseed. Fermentation. (2023) 9:522. doi: 10.3390/fermentation9060522

[39] Herkenhoff ME de Medeiros IUD Garutti LHG Salgaco MK Sivieri K Saad SMI. Cashew by-product as a functional substrate for the development of probiotic fermented Milk. Foods. (2023) 12:3383. doi: 10.3390/foods12183383, PMID: 37761092 PMC10528859

[40] Luz C Calpe J Manuel Quiles J Torrijos R Vento M Gormaz M . Probiotic characterization of *Lactobacillus* strains isolated from breast milk and employment for the elaboration of a fermented milk product. J Funct Foods. (2021) 84:4599. doi: 10.1016/j.jff.2021.104599

[41] Aziz T Xingyu H Sarwar A Naveed M Shabbir MA Khan AA . Assessing the probiotic potential, antioxidant, and antibacterial activities of oat and soy milk fermented with Lactiplantibacillus plantarum strains isolated from Tibetan kefir. Front Microbiol. (2023) 14:1265188. doi: 10.3389/fmicb.2023.1265188, PMID: 37817753 PMC10560984

[42] Abdelshafy AM Rashwan AK Osman AI. Potential food applications and biological activities of fermented quinoa: a review. Trend Food Sci Technol. (2024) 144:1–15. doi: 10.1016/j.tifs.2024.104339

[43] Shrushti Makwana JB Prajapati PR Hati S. Effects of probiotic fermented milk on management of obesity studied in high-fat-diet induced obese rat model. Food Prod Process Nutr. (2023) 5:1–18. doi: 10.1186/s43014-023-00112-1

[44] Parmar U Sreeja V Kiran S Sandhya H Jakhesara S. Anticancer effect of postbiotic derived from fermented milk of *Lactobacillus helveticus* MTCC 5463 on HT-29: anticancer effect of fermented milk postbiotic. Indian J Exper Biol. (2025) 63:312–22. doi: 10.56042/ijeb.v63i04.11559

[45] Freitas SM Franco B Saragiotto G Morais MA Simabuco FM Cunha DT . Effect of a probiotic fermented milk supplementation on behavior and sleep. Nutr Neurosci. (2023) 27:1–13. doi: 10.1080/1028415X.2023.2240990 37496309

[46] Zambrano-Cervantes M Gonzalez-Cordova AF Hernandez-Mendoza A Beltran-Barrientos LM Rendon-Rosales MA Manzanarez-Quin CG . Fermented milks with specific Lactobacillus spp. with potential cardioprotective effects. J Food Sci Technol. (2023) 60:1749–60. doi: 10.1007/s13197-023-05715-1, PMID: 37179799 PMC10122198

[47] Mantel M da Silva TF Gloria R Vassaux D Vital KD Cardoso VN . Fat matters: fermented whole milk potentiates the anti-colitis effect of *Propionibacterium freudenreichii*. J Funct Foods. (2023) 106. doi: 10.1016/j.jff.2023.105614

[48] Guo Z-H Wang Q Zhao J-H Xu Y-P Mu G-Q Zhu X-M. Lactic acid bacteria with probiotic characteristics in fermented dairy products reduce cow milk allergy. Food Bioscience. (2023) 55. doi: 10.1016/j.fbio.2023.103055

[49] Santiago-López L Hernández-Mendoza A Vallejo-Cordoba B Wall-Medrano A González-Córdova AF. Th17 immune response in inflammatory bowel disease: future roles and opportunities for lactic acid bacteria and bioactive compounds released in fermented milk. Trends Food Sci Technol. (2021) 112:109–17. doi: 10.1016/j.tifs.2021.03.051

[50] Yang S Bai M Kwok LY Zhong Z Sun Z. The intricate symbiotic relationship between lactic acid bacterial starters in the milk fermentation ecosystem. Crit Rev Food Sci Nutr. (2023) 65:1–18. doi: 10.1080/10408398.2023.2280706 37983125

[51] Vasudha M Prashantkumar CS Bellurkar M Kaveeshwar V Gayathri D. Probiotic potential of beta-galactosidase-producing lactic acid bacteria from fermented milk and their molecular characterization. Biomed Rep. (2023) 18:23. doi: 10.3892/br.2023.1605 36846619 PMC9945298

[52] Galli V Venturi M Mari E Guerrini S Granchi L. Gamma-aminobutyric acid (GABA) production in fermented milk by lactic acid bacteria isolated from spontaneous raw milk fermentation. Int Dairy J. (2022) 127:5284. doi: 10.1016/j.idairyj.2021.105284

[53] Yu L Han X Cen S Duan H Feng S Xue Y . Beneficial effect of GABA-rich fermented milk on insomnia involving regulation of gut microbiota. Microbiol Res. (2020) 233:126409. doi: 10.1016/j.micres.2020.126409, PMID: 31927503

[54] Codex. CODEX STAN 243–2003: Standard for fermented milks (revised in 2010 ed., Vol. 2012). (2003).

[55] Nielsen SD Jakobsen LMA Geiker NRW Bertram HC. Chemically acidified, live and heat-inactivated fermented dairy yoghurt show distinct bioactive peptides, free amino acids and small compounds profiles. Food Chem. (2021) 376:131919. doi: 10.1016/j.foodchem.2021.131919 34968909

[56] Hayes E Wallace D O'Donnell C Greene D Hennessy D O'Shea N . Trend analysis and prediction of seasonal changes in milk composition from a pasture-based dairy research herd. J Dairy Sci. (2023) 106:2326–37. doi: 10.3168/jds.2021-21483, PMID: 36759275

[57] Rehman H Saipriya K Singh AK Singh R Meena GS Khetra Y . A metabolomics approach to establish the relationship between the techno-functional properties and metabolome of Indian goat yoghurt. Foods. (2024) 13:913. doi: 10.3390/foods13060913, PMID: 38540903 PMC10969914

[58] Shi C Maktabdar M. Lactic acid Bacteria as biopreservation against spoilage molds in dairy products-a review. Front Microbiol. (2021) 12:819684. doi: 10.3389/fmicb.2021.819684 35154045 PMC8826399

[59] Sharma H El Rassi GD Lathrop A Dobreva VB Belem TS Ramanathan R. Comparative analysis of metabolites in cow and goat milk yoghurt using GC–MS based untargeted metabolomics. Int Dairy J. (2021) 117:105016. doi: 10.1016/j.idairyj.2021.105016

[60] Murgia A Scano P Cacciabue R Dessì D Caboni P. Gc-ms metabolomics comparison of yoghurts from sheep's and goats' milk. Int Dairy J. (2019) 96:44–9. doi: 10.1016/j.idairyj.2019.03.012

[61] Trimigno A Bøge Lyndgaard C Atladóttir GA Aru V Balling Engelsen S Harder Clemmensen LK. An NMR metabolomics approach to investigate factors affecting the yoghurt fermentation process and quality. Meta. (2020) 10:293. doi: 10.3390/metabo10070293, PMID: 32709034 PMC7408429

[62] Pannerchelvan S Rios-Solis L Wasoh H Sobri MZM Wong FWF Mohamed MS . Functional yogurt: a comprehensive review of its nutritional composition and health benefits. Food Funct. (2024). doi: 10.1039/D4FO03671A 39446126

[63] Dan T Chen H Li T Tian J Ren W Zhang H . Influence of *Lactobacillus plantarum* P-8 on fermented Milk flavor and storage stability. Front Microbiol. (2018) 9:3133. doi: 10.3389/fmicb.2018.03133 30687239 PMC6333906

[64] Wang J Zhao W Guo S Sun Y Yao K Liu Z . Different growth behaviors and metabolomic profiles in yogurts induced by multistrain probiotics of *Lactobacillus casei* Zhang and *Bifidobacterium lactis* V9 under different fermentation temperatures. J Dairy Sci. (2021) 104:10528–39. doi: 10.3168/jds.2021-20352, PMID: 34334203

[65] González S Fernández-Navarro T Arboleya S de Los Reyes-Gavilán C Salazar N Gueimonde M. Fermented dairy foods: impact on intestinal microbiota and health-linked biomarkers. Front Microbiol. (2019) 10:1046. doi: 10.3389/fmicb.2019.01046, PMID: 31191465 PMC6545342

[66] Uebanso T Ohnishi A Kitayama R Yoshimoto A Nakahashi M Shimohata T . Effects of low-dose non-caloric sweetener consumption on gut microbiota in mice. Nutrients. (2017) 9:560. doi: 10.3390/nu9060560, PMID: 28587159 PMC5490539

[67] Mu W Chen Q Wang X Zhang T Jiang B. Current studies on physiological functions and biological production of lactosucrose. Appl Microbiol Biotechnol. (2013) 97:7073–80. doi: 10.1007/s00253-013-5079-3, PMID: 23828605

[68] Xue R Liu J Zhang M Aziz T Felemban S Khowdiary MM . Physicochemical, microbiological and metabolomics changes in yogurt supplemented with lactosucrose. Food Res Int. (2024) 178:114000. doi: 10.1016/j.foodres.2024.114000, PMID: 38309926

[69] Tutunchi H Naghshi S Naemi M Naeini F Esmaillzadeh A. Yogurt consumption and risk of mortality from all causes, CVD and cancer: a comprehensive systematic review and dose-response meta-analysis of cohort studies. Public Health Nutr. (2023) 26:1196–209. doi: 10.1017/S1368980022002385, PMID: 36349966 PMC10346031

[70] Bütikofer U Badertscher R Blaser-Freiburghaus C Fuchsmann P Tena Stern M Kuert PA . Serum and urine metabolites in healthy men after consumption of acidified Milk and yogurt. Nutrients. (2022) 14:4794. doi: 10.3390/nu14224794, PMID: 36432479 PMC9698558

[71] Du S Chen Y Liu X Zhang Z Jiang Y Zhou Y . Two untargeted metabolomics reveals yogurt-associated metabolic alterations in women with multiple metabolic disorders from a randomized controlled study. J Proteome. (2022) 252:104394. doi: 10.1016/j.jprot.2021.104394, PMID: 34666202

[72] Correia BSB Sandby K Krarup T Magkos F Geiker NRW Bertram HC. Changes in plasma, urine, and fecal metabolome after 16 weeks of consuming dairy with different food matrixes – a randomized controlled trial. Mol Nutr Food Res. (2024) 68:e2300363. doi: 10.1002/mnfr.202300363, PMID: 38299443

[73] Kim J Blaser C Portmann R Badertscher R Marmonier C Blot A . Postprandial responses on serum metabolome to Milk and yogurt intake in young and older men. Front Nutr. (2022) 9:851931. doi: 10.3389/fnut.2022.851931, PMID: 35600812 PMC9115859

[74] Papadimitriou K Georgalaki M Anastasiou R Alexandropoulou A-M Manolopoulou E Zoumpopoulou G . Study of the microbiome of the cretan sour cream staka using amplicon sequencing and shotgun metagenomics and isolation of novel strains with an important antimicrobial potential. Foods. (2024) 13:1129. doi: 10.3390/foods13071129, PMID: 38611432 PMC11011300

[75] Yu J Mo L Pan L Yao C Ren D An X . Bacterial microbiota and metabolic character of traditional sour cream and butter in Buryatia, Russia. Front Microbiol. (2018) 9:2496. doi: 10.3389/fmicb.2018.02496, PMID: 30459729 PMC6232932

[76] Katke S Rahman M Patil P. Standardization and quality evaluation of sour cream enriched therapeutic food products. Int J Curr Microbiol Appl Sci. (2019) 8:1449–61. doi: 10.20546/ijcmas.2019.803.169

[77] Shepard L Miracle R Leksrisompong P Drake M. Relating sensory and chemical properties of sour cream to consumer acceptance. J Dairy Sci. (2013) 96:5435–54. doi: 10.3168/jds.2012-6317, PMID: 23849637

[78] Yu J Wang H Zha M Qing Y Bai N Ren Y . Molecular identification and quantification of lactic acid bacteria in traditional fermented dairy foods of Russia. J Dairy Sci. (2015) 98:5143–54. doi: 10.3168/jds.2015-9460, PMID: 26004836

[79] Kondrotiene K Zavistanaviciute P Aksomaitiene J Novoslavskij A Malakauskas M. *Lactococcus lactis* in dairy fermentation—health-promoting and probiotic properties. Fermentation. (2023) 10:16. doi: 10.3390/fermentation10010016

[80] Jung MY Lee C Seo M-J Roh SW Lee SH. Characterization of a potential probiotic bacterium *Lactococcus raffinolactis* WiKim0068 isolated from fermented vegetable using genomic and in vitro analyses. BMC Microbiol. (2020) 20:136. doi: 10.1186/s12866-020-01820-9, PMID: 32460704 PMC7251713

[81] Gao J Li X Zhang G Sadiq FA Simal-Gandara J Xiao J . Probiotics in the dairy industry—advances and opportunities. Compr Rev Food Sci Food Saf. (2021) 20:3937–82. doi: 10.1111/1541-4337.12755, PMID: 33938124

[82] Lee SH Jung JY Jeon CO. Bacterial community dynamics and metabolite changes in myeolchi-aekjeot, a Korean traditional fermented fish sauce, during fermentation. Int J Food Microbiol. (2015) 203:15–22. doi: 10.1016/j.ijfoodmicro.2015.02.031, PMID: 25770429

[83] Jung JY Lee HJ Chun BH Jeon CO. Effects of temperature on bacterial communities and metabolites during fermentation of myeolchi-aekjeot, a traditional Korean fermented anchovy sauce. PLoS One. (2016) 11:e0151351. doi: 10.1371/journal.pone.0151351, PMID: 26977596 PMC4792383

[84] Gaba K Anand S. Incorporation of probiotics and other functional ingredients in dairy fat-rich products: benefits, challenges, and opportunities. Dairy. (2023) 4:630–49. doi: 10.3390/dairy4040044

[85] Khademi F Raeisi SN Younesi M Motamedzadegan A Rabiei K Shojaei M . Effect of probiotic bacteria on physicochemical, microbiological, textural, sensory properties and fatty acid profile of sour cream. Food Chem Toxicol. (2022) 166:113244. doi: 10.1016/j.fct.2022.113244, PMID: 35728727

[86] Hansson P Holven KB Øyri LK Brekke HK Biong AS Gjevestad GO . Meals with similar fat content from different dairy products induce different postprandial triglyceride responses in healthy adults: a randomized controlled cross-over trial. J Nutr. (2019) 149:422–31. doi: 10.1093/jn/nxy291, PMID: 30759235 PMC6398384

[87] Keirns BH Sciarrillo CM Koemel NA Emerson SR. Fasting, non-fasting and postprandial triglycerides for screening cardiometabolic risk. J Nutr Sci. (2021) 10:e75. doi: 10.1017/jns.2021.73, PMID: 34589207 PMC8453457

[88] Rathnayake KM Weech M Jackson KG Lovegrove JA. Impact of meal fatty acid composition on postprandial lipaemia, vascular function and blood pressure in postmenopausal women. Nutr Res Rev. (2018) 31:193–203. doi: 10.1017/S0954422418000033, PMID: 29547370

[89] Calvo MV Martín-Hernández MC García-Serrano A Castro-Gómez MP Alonso-Miravalles L García-Martín R . Comprehensive characterization of neutral and polar lipids of buttermilk from different sources and its milk fat globule membrane isolates. J Food Compos Anal. (2020) 86:103386. doi: 10.1016/j.jfca.2019.103386

[90] Conway V Gauthier S Pouliot Y. Buttermilk: much more than a source of milk phospholipids. Anim Front. (2014) 4:44–51. doi: 10.2527/af.2014-001432288969

[91] Azarcoya-Barrera J Field CJ Goruk S Makarowski A Curtis JM Pouliot Y . Buttermilk: an important source of lipid soluble forms of choline that influences the immune system development in Sprague–Dawley rat offspring. Eur J Nutr. (2021) 60:1–12. doi: 10.1007/s00394-020-02462-3 33416979

[92] Rombaut R Camp JV Dewettinck K. Phospho-and sphingolipid distribution during processing of milk, butter and whey. Int J Food Sci Technol. (2006) 41:435–43. doi: 10.1111/j.1365-2621.2005.01091.x

[93] Morin P Jiménez-Flores R Pouliot Y. Effect of processing on the composition and microstructure of buttermilk and its milk fat globule membranes. Int Dairy J. (2007) 17:1179–87. doi: 10.1016/j.idairyj.2007.03.010

[94] Señoráns M Gallo V Calvo MV Fontecha J. Lipidomic and proteomic profiling of the milk fat globule membrane from different industrial by-products of the butter and butter oil manufacturing process. Foods. (2023) 12:750. doi: 10.3390/foods12040750, PMID: 36832824 PMC9956092

[95] Holzmüller W Kulozik U. Quantification of MFGM proteins in buttermilk and butter serum by means of a stain free SDS-PAGE method. J Food Compos Anal. (2016) 49:102–9. doi: 10.1016/j.jfca.2016.04.003

[96] Ali AH. Current knowledge of buttermilk: composition, applications in the food industry, nutritional and beneficial health characteristics. Int J Dairy Technol. (2019) 72:169–82. doi: 10.1111/1471-0307.12572

[97] Raza GS Herzig K-H Leppäluoto J. Invited review: Milk fat globule membrane—a possible panacea for neurodevelopment, infections, cardiometabolic diseases, and frailty. J Dairy Sci. (2021) 104:7345–63. doi: 10.3168/jds.2020-19649, PMID: 33896625

[98] Conway V Gauthier SF Pouliot Y. Effect of cream pasteurization, microfiltration and enzymatic proteolysis on in vitro cholesterol-lowering activity of buttermilk solids. Dairy Sci Technol. (2010) 90:449–60. doi: 10.1051/dst/2010021

[99] Conway V Couture P Richard C Gauthier S Pouliot Y Lamarche B. Impact of buttermilk consumption on plasma lipids and surrogate markers of cholesterol homeostasis in men and women. Nutr Metab Cardiovasc Dis. (2013) 23:1255–62. doi: 10.1016/j.numecd.2013.03.003, PMID: 23786821

[100] Baumgartner S Kelly ER van der Made S Berendschot TT Husche C Lütjohann D . The influence of consuming an egg or an egg-yolk buttermilk drink for 12 wk on serum lipids, inflammation, and liver function markers in human volunteers. Nutrition. (2013) 29:1237–44. doi: 10.1016/j.nut.2013.03.020, PMID: 23911216

[101] Yeragi V Maske A. Effects of buttermilk on health. Int J Scient Res Manag. (2016) 4:4936.

[102] Kuchta-Noctor AM Murray BA Stanton C Devery R Kelly PM. Anticancer activity of buttermilk against SW480 colon cancer cells is associated with caspase-independent cell death and attenuation of Wnt, Akt, and ERK signaling. Nutr Cancer. (2016) 68:1234–46. doi: 10.1080/01635581.2016.1206580, PMID: 27472445

[103] Conway V Couture P Gauthier S Pouliot Y Lamarche B. Effect of buttermilk consumption on blood pressure in moderately hypercholesterolemic men and women. Nutrition. (2014) 30:116–9. doi: 10.1016/j.nut.2013.07.021, PMID: 24206823

[104] Bourlieu C Cheillan D Blot M Daira P Trauchessec M Ruet S . Polar lipid composition of bioactive dairy co-products buttermilk and butterserum: emphasis on sphingolipid and ceramide isoforms. Food Chem. (2018) 240:67–74. doi: 10.1016/j.foodchem.2017.07.091, PMID: 28946327

[105] Guzel-Seydim ZB Gökırmaklı Ç Greene AK. A comparison of milk kefir and water kefir: physical, chemical, microbiological and functional properties. Trends Food Sci Technol. (2021) 113:42–53. doi: 10.1016/j.tifs.2021.04.041

[106] Esener OBB Balkan B Armutak E Uvez A Yildiz G Hafizoglu M . Donkey milk kefir induces apoptosis and suppresses proliferation of Ehrlich ascites carcinoma by decreasing iNOS in mice. Biotech Histochem. (2018) 93:424–31. doi: 10.1080/10520295.2018.1448112, PMID: 29642726

[107] Magalhães-Guedes K Barreto I Tavares P Bezerra P Silva M Nunes I . Effect of kefir biomass on nutritional, microbiological, and sensory properties of mango-based popsicles. Int Food Res J. (2020) 27:536–45.

[108] Azizi NF Kumar MR Yeap SK Abdullah JO Khalid M Omar AR . Kefir and its biological activities. Foods. (2021) 10:1210. doi: 10.3390/foods10061210 34071977 PMC8226494

[109] Garofalo C Ferrocino I Reale A Sabbatini R Milanović V Alkić-Subašić M . Study of kefir drinks produced by backslopping method using kefir grains from Bosnia and Herzegovina: microbial dynamics and volatilome profile. Food Res Int. (2020) 137:109369. doi: 10.1016/j.foodres.2020.109369, PMID: 33233071

[110] Nejati F Capitain CC Krause JL Kang G-U Riedel R Chang H-D . Traditional grain-based vs. commercial Milk kefirs, how different are they? Appl Sci. (2022) 12:3838. doi: 10.3390/app12083838

[111] Vieira CP Rosario AIL Lelis CA Rekowsky BSS Carvalho APA Rosário DKA . Bioactive compounds from kefir and their potential benefits on health: a systematic review and meta-analysis. Oxidative Med Cell Longev. (2021) 2021:1738. doi: 10.1155/2021/9081738, PMID: 34745425 PMC8566050

[112] Guangsen T Xiang L Jiahu G. Microbial diversity and volatile metabolites of kefir prepared by different milk types. CyTA J Food. (2021) 19:399–407. doi: 10.1080/19476337.2021.1912190

[113] Fiorda FA de Melo Pereira GV Thomaz-Soccol V Rakshit SK Pagnoncelli MGB de Souza Vandenberghe LP . Microbiological, biochemical, and functional aspects of sugary kefir fermentation-a review. Food Microbiol. (2017) 66:86–95. doi: 10.1016/j.fm.2017.04.004, PMID: 28576377

[114] Prado MR Blandón LM Vandenberghe LP Rodrigues C Castro GR Thomaz-Soccol V . Milk kefir: composition, microbial cultures, biological activities, and related products. Front Microbiol. (2015) 6:1177. doi: 10.3389/fmicb.2015.01177 26579086 PMC4626640

[115] Syrokou MK Papadelli M Ntaikou I Paramithiotis S Drosinos EH. Sugary kefir: microbial identification and biotechnological properties. Beverages. (2019) 5:61. doi: 10.3390/beverages5040061

[116] Kurmann JA Rasic JL Kroger M. Encyclopedia of fermented fresh milk products: An international inventory of fermented milk, cream, buttermilk, whey, and related products. Amsterdam, Netherlands: Springer Science & Business Media (1992).

[117] Tiss M Souiy Z ben Abdeljelil N Njima M Achour L Hamden K. Fermented soy milk prepared using kefir grains prevents and ameliorates obesity, type 2 diabetes, hyperlipidemia and liver-kidney toxicities in HFFD-rats. J Funct Foods. (2020) 67:103869. doi: 10.1016/j.jff.2020.103869

[118] Erdogan FS Ozarslan S Guzel-Seydim ZB Taş TK. The effect of kefir produced from natural kefir grains on the intestinal microbial populations and antioxidant capacities of Balb/c mice. Food Res Int. (2019) 115:408–13. doi: 10.1016/j.foodres.2018.10.080, PMID: 30599959

[119] Jeong D Kim D-H Kang I-B Kim H Song K-Y Kim H-S . Characterization and antibacterial activity of a novel exopolysaccharide produced by *Lactobacillus kefiranofaciens* DN1 isolated from kefir. Food Control. (2017) 78:436–42. doi: 10.1016/j.foodcont.2017.02.033

[120] Cui Y Wang X Yue Y Du G Chen H Ning M . Metagenomic features of Tibetan kefir grains and its metabolomics analysis during fermentation. LWT. (2023) 175:114502. doi: 10.1016/j.lwt.2023.114502

[121] Malta SM Batista LL Silva HCG Franco RR Silva MH Rodrigues TS . Identification of bioactive peptides from a Brazilian kefir sample, and their anti-Alzheimer potential in *Drosophila melanogaster*. Sci Rep. (2022) 12:11065. doi: 10.1038/s41598-022-15297-1, PMID: 35773306 PMC9246878

[122] Wang H Zhou X Sun Y Sun X Guo M. Differences in protein profiles of kefir grains from different origins when subcultured in goat Milk. J Agric Food Chem. (2022) 70:7515–24. doi: 10.1021/acs.jafc.2c01391, PMID: 35687069

[123] Bourrie BC Diether N Dias RP Nam SL de la Mata AP Forgie AJ . Use of reconstituted kefir consortia to determine the impact of microbial composition on kefir metabolite profiles. Food Res Int. (2023) 173:113467. doi: 10.1016/j.foodres.2023.113467 37803789

[124] de Oliveira Filho JG de Oliveira Silva C Egea MB de Azeredo HMC Mattoso LHC. Employing alternative culture media in kefiran exopolysaccharide production: impact on microbial diversity, physicochemical properties, and bioactivities. Int J Biol Macromol. (2023) 246:125648. doi: 10.1016/j.ijbiomac.2023.125648 37406922

[125] Seo K-H Lee HG Eor JY Jeon HJ Yokoyama W Kim H. Effects of kefir lactic acid bacteria-derived postbiotic components on high fat diet-induced gut microbiota and obesity. Food Res Int. (2022) 157:111445. doi: 10.1016/j.foodres.2022.111445, PMID: 35761685

[126] Albuquerque Pereira MF de Morais Ávila LG Ávila Alpino GC dos Santos Cruz BC Almeida LF Macedo Simões J . Milk kefir alters fecal microbiota impacting gut and brain health in mice. Appl Microbiol Biotechnol. (2023) 107:5161–78. doi: 10.1007/s00253-023-12630-0 37389589

[127] Blasche S Kim Y Mars RA Machado D Maansson M Kafkia E . Metabolic cooperation and spatiotemporal niche partitioning in a kefir microbial community. Nat Microbiol. (2021) 6:196–208. doi: 10.1038/s41564-020-00816-5, PMID: 33398099 PMC7610452

[128] Guo C Chen Y Wu D Du Y Wang M Liu C . Transcriptome analysis reveals an essential role of exogenous brassinolide on the alkaloid biosynthesis pathway in *Pinellia Ternata*. Int J Mol Sci. (2022) 23:10898. doi: 10.3390/ijms231810898, PMID: 36142812 PMC9501358

[129] Bengoa AA Iraporda C Garrote GL Abraham AG. Kefir micro-organisms: their role in grain assembly and health properties of fermented milk. J Appl Microbiol. (2019) 126:686–700. doi: 10.1111/jam.14107, PMID: 30218595

[130] Ponomarova O Gabrielli N Sévin DC Mülleder M Zirngibl K Bulyha K . Yeast creates a niche for symbiotic lactic acid bacteria through nitrogen overflow. Cell Syst. (2017) 5:345–357.e6. e6. doi: 10.1016/j.cels.2017.09.002, PMID: 28964698 PMC5660601

[131] Ebner J Arslan AA Fedorova M Hoffmann R Küçükçetin A Pischetsrieder M. Peptide profiling of bovine kefir reveals 236 unique peptides released from caseins during its production by starter culture or kefir grains. J Proteome. (2015) 117:41–57. doi: 10.1016/j.jprot.2015.01.005, PMID: 25613046

[132] Quirós A Hernández-Ledesma B Ramos M Amigo L Recio I. Angiotensin-converting enzyme inhibitory activity of peptides derived from caprine kefir. J Dairy Sci. (2005) 88:3480–7. doi: 10.3168/jds.S0022-0302(05)73032-0, PMID: 16162521

[133] Akar F Sumlu E Alçığır ME Bostancı A Sadi G. Potential mechanistic pathways underlying intestinal and hepatic effects of kefir in high-fructose-fed rats. Food Res Int. (2021) 143:110287. doi: 10.1016/j.foodres.2021.110287, PMID: 33992387

[134] De LeBlanc ADM Matar C Farnworth E Perdigón G. Study of immune cells involved in the antitumor effect of kefir in a murine breast cancer model. J Dairy Sci. (2007) 90:1920–8. doi: 10.3168/jds.2006-079 17369232

[135] Du G Liu L Guo Q Cui Y Chen H Yuan Y . Microbial community diversity associated with Tibetan kefir grains and its detoxification of Ochratoxin a during fermentation. Food Microbiol. (2021) 99:103803. doi: 10.1016/j.fm.2021.103803, PMID: 34119096

[136] Tenorio-Salgado S Castelán-Sánchez H Dávila-Ramos S Huerta-Saquero A Rodríguez-Morales S de la Fuente R . Metagenomic analysis and antimicrobial activity of two fermented milk kefir samples. Microbiol Open. (2021) 10:e1183. doi: 10.1002/mbo3.1183 PMC8103080 33970536

[137] Gao J Ding G Li Q Gong L Huang J Sang Y. Tibet kefir milk decreases fat deposition by regulating the gut microbiota and gene expression of Lpl and Angptl4 in high fat diet-fed rats. Food Res Int. (2019) 121:278–87. doi: 10.1016/j.foodres.2019.03.029, PMID: 31108749

[138] Santini G Bonazza F Pucciarelli S Polidori P Ricciutelli M Klimanova Y . Proteomic characterization of kefir milk by two-dimensional electrophoresis followed by mass spectrometry. J Mass Spectrom. (2020) 55:e4635. doi: 10.1002/jms.4635, PMID: 32767505

[139] Izquierdo-González JJ Amil-Ruiz F Zazzu S Sánchez-Lucas R Fuentes-Almagro CA Rodríguez-Ortega MJ. Proteomic analysis of goat milk kefir: profiling the fermentation-time dependent protein digestion and identification of potential peptides with biological activity. Food Chem. (2019) 295:456–65. doi: 10.1016/j.foodchem.2019.05.178, PMID: 31174782

[140] Ventura G Bianco M Losito I Cataldi TR Calvano CD. Complete polar lipid profile of kefir beverage by hydrophilic interaction liquid chromatography with HRMS and tandem mass spectrometry. Int J Mol Sci. (2025) 26:1120. doi: 10.3390/ijms26031120, PMID: 39940887 PMC11818909

[141] Afzaal M Saeed F Anjum F Waris N Husaain M Ikram A . Nutritional and ethnomedicinal scenario of koumiss: a concurrent review. Food Sci Nutr. (2021) 9:6421–8. doi: 10.1002/fsn3.2595, PMID: 34760271 PMC8565204

[142] XiLin T He X-L Bi Y-Q Gao Y Chen A Urtnasan M . Research progress on chemical composition, microbial diversity and effects on human health of koumiss. Food Med Homol. (2025) 2:27. doi: 10.26599/FMH.2025.9420027

[143] Yaman H Aykas DP Rodriguez-Saona LE. Monitoring Turkish white cheese ripening by portable FT-IR spectroscopy. Front Nutr. (2023) 10:1107491. doi: 10.3389/fnut.2023.1107491, PMID: 36814504 PMC9940898

[144] Bettera L Levante A Bancalari E Bottari B Gatti M. Lactic acid bacteria in cow raw milk for cheese production: which and how many? Front Microbiol. (2023) 13:1092224. doi: 10.3389/fmicb.2022.1092224, PMID: 36713157 PMC9878191

[145] Cao W Aubert J Maillard M-B Boissel F Leduc A Thomas J-L . Fine-tuning of process parameters modulates specific metabolic bacterial activities and aroma compound production in semi-hard cheese. J Agric Food Chem. (2021) 69:8511–29. doi: 10.1021/acs.jafc.1c01634, PMID: 34283609

[146] Tarnaud F Gaucher F Do Carmo FLR Illikoud N Jardin J Briard-Bion V . Differential adaptation of *Propionibacterium freudenreichii* CIRM-BIA129 to cow’s Milk versus soymilk environments modulates its stress tolerance and proteome. Front Microbiol. (2020) 11:549027. doi: 10.3389/fmicb.2020.549027, PMID: 33335514 PMC7736159

[147] Afshari R Pillidge CJ Dias DA Osborn AM Gill H. Biomarkers associated with cheese quality uncovered by integrative multi-omic analysis. Food Control. (2021) 123:107752. doi: 10.1016/j.foodcont.2020.107752

[148] Ertekin M Uğurlu Ö Salum P Erbay Z. Effects of milk types used in Antep cheese production on some cheese organoleptic quality parameters and brine composition during 5-month ripening. J Food Sci. (2023) 88:1445–65. doi: 10.1111/1750-3841.16519, PMID: 36877142

[149] Tekin A Hayaloglu AA. Understanding the mechanism of ripening biochemistry and flavour development in brine ripened cheeses. Int Dairy J. (2023) 137:105508. doi: 10.1016/j.idairyj.2022.105508

[150] Tomita S Nomura M Arakawa Y Miura T Hayashida S Hagi T . Volatile and soluble metabolite profiles in surface-ripened cheeses with aspergillus oryzae and aspergillus sojae. Food Res Int. (2022) 158:111535. doi: 10.1016/j.foodres.2022.111535, PMID: 35840232

[151] Lecomte M Cao W Aubert J Sherman DJ Falentin H Frioux C . A digital twin of bacterial metabolism during cheese production. (2023). [Epubh ahead of preprint]. doi: 10.1101/2023.05.05.539417

[152] Liu J-M Chen L Dorau R Lillevang SK Jensen PR Solem C. From waste to taste—efficient production of the butter aroma compound acetoin from low-value dairy side streams using a natural (nonengineered) *Lactococcus lactis* dairy isolate. J Agric Food Chem. (2020) 68:5891–9. doi: 10.1021/acs.jafc.0c00882, PMID: 32363876

[153] McAuliffe O Kilcawley K Stefanovic E. Symposium review: genomic investigations of flavor formation by dairy microbiota. J Dairy Sci. (2019) 102:909–22. doi: 10.3168/jds.2018-15385, PMID: 30343908

[154] Kocak A Sanli T Anli EA Hayaloglu AA. Role of using adjunct cultures in release of bioactive peptides in white-brined goat-milk cheese. LWT. (2020) 123:109127. doi: 10.1016/j.lwt.2020.109127

[155] Kochetkova TV Grabarnik IP Klyukina AA Zayulina KS Gavirova LA Shcherbakova PA . The bacterial microbiota of artisanal cheeses from the northern Caucasus. Fermentation. (2023) 9:719. doi: 10.3390/fermentation9080719

[156] Martín I Rodríguez A Alía A Martínez-Blanco M Lozano-Ojalvo D Córdoba JJ. Control of *Listeria monocytogenes* growth and virulence in a traditional soft cheese model system based on lactic acid bacteria and a whey protein hydrolysate with antimicrobial activity. Int J Food Microbiol. (2022) 361:109444. doi: 10.1016/j.ijfoodmicro.2021.109444, PMID: 34749186

[157] Gasser BG Fuchsmann P Fröhlich-Wyder MT. Sensory characteristics of Swiss-type cheese varieties In: Sensory profiling of dairy products (New Jersey, USA: John Wiley & Sons Ltd.) (2023) 195–224.

[158] Anastasiou R Kazou M Georgalaki M Aktypis A Zoumpopoulou G Tsakalidou E. Omics approaches to assess flavor development in cheese. Foods. (2022) 11:188. doi: 10.3390/foods11020188, PMID: 35053920 PMC8775153

[159] Garofalo G Taspinar T Busetta G Mastrangelo S Portolano B Sardina MT . Description of Ewiss cheese, a new ewe milk cheese processed by Swiss cheese manufacturing techniques: microbiological, physicochemical, and sensory aspects. J Dairy Sci. (2024) 107:6614–28. doi: 10.3168/jds.2024-24711, PMID: 38754834

[160] Hayashida S Hagi T Kobayashi M Kusumoto K-I Ohmori H Tomita S . Comparison of taste characteristics between koji mold–ripened cheese and camembert cheese using an electronic tongue system. J Dairy Sci. (2023) 106:6701–9. doi: 10.3168/jds.2023-23277, PMID: 37210348

[161] Bates M Clark S. Mold-ripened cheeses. The Sensory Evaluation of Dairy Products. Amsterdam, Netherlands: Springer (2023). 545–570.

[162] Bodinaku I Shaffer J Connors AB Steenwyk JL Biango-Daniels MN Kastman EK . Rapid phenotypic and metabolomic domestication of wild Penicillium molds on cheese. MBio. (2019) 10:1–16. doi: 10.1128/mbio.02445-19 PMC6794487 31615965

[163] Bertuzzi AS Walsh AM Sheehan J Cotter PD Crispie F McSweeney PL . Omics-based insights into flavor development and microbial succession within surface-ripened cheese. MSystems. (2018) 3:1–17. doi: 10.1128/msystems00211-17 PMC5790873 29404426

[164] Unno R Suzuki T Matsutani M Ishikawa M. Evaluation of the relationships between microbiota and metabolites in soft-type ripened cheese using an integrated omics approach. Front Microbiol. (2021) 12:681185. doi: 10.3389/fmicb.2021.681185, PMID: 34168634 PMC8219077

[165] Panthi RR Kelly AL O'Callaghan DJ Sheehan JJ. Measurement of syneretic properties of rennet-induced curds and impact of factors such as concentration of milk: a review. Trends Food Sci Technol. (2019) 91:530–40. doi: 10.1016/j.tifs.2019.07.023

[166] Caswell EN. Effects of microbial lipases on parmesan and feta cheese flavor profiles. (2024).

[167] Becchi PP Rocchetti G García-Pérez P Michelini S Pizzamiglio V Lucini L. Untargeted metabolomics and machine learning unveil quality and authenticity interactions in grated Parmigiano Reggiano PDO cheese. Food Chem. (2024) 447:138938. doi: 10.1016/j.foodchem.2024.138938, PMID: 38458130

[168] Farsi DN Mathur H Beresford T Cotter PD. Cottage cheese, a relatively underexplored cultured dairy product with potential health benefits? Crit Rev Food Sci Nutr. (2025) 6:1–11. doi: 10.1080/10408398.2025.2487682 40188423

[169] Ma Q Liu L Jiao Y Qiao X Han R Li X . Insights into flavor quality and metabolites profiles of fresh cheese with different probiotics by SPME-GC-MS and untargeted metabolomics. Food Res Int. (2024) 197:115154. doi: 10.1016/j.foodres.2024.115154, PMID: 39593366

[170] Milanović SD Hrnjez DV Iličić MD Kanurić KG Vukić VR. (2016):165–201. Novel fermented dairy products. Novel Food Fermentation Technologies. (Amsterdam, Holland: Springer).

[171] Adam AC Rubio-Texeira M Polaina J. Lactose: the milk sugar from a biotechnological perspective. BFSN. (2005) 44:553–7. doi: 10.1080/10408690490931411 15969327

[172] Park YW Haenlein GF. Milk and dairy products in human nutrition: Production, composition and health. New Jersey, USA: John Wiley & Sons (2013).

[173] Tomar BS. Lactose intolerance and other disaccharidase deficiency. Indian J Pediatr. (2014) 81:876–80. doi: 10.1007/s12098-014-1346-2, PMID: 24596060

[174] Li A Zheng J Han X Jiang Z Yang B Yang S . Health implication of lactose intolerance and updates on its dietary management. Int Dairy J. (2023) 140:105608. doi: 10.1016/j.idairyj.2023.105608

[175] Sharp E D'Cunha NM Ranadheera CS Vasiljevic T Panagiotakos DB Naumovski N. Effects of lactose-free and low-lactose dairy on symptoms of gastrointestinal health: a systematic review. Int Dairy J. (2021) 114:104936. doi: 10.1016/j.idairyj.2020.104936

[176] Storhaug CL Fosse SK Fadnes LT. Country, regional, and global estimates for lactose malabsorption in adults: a systematic review and meta-analysis. Lancet Gastroenterol Hepatol. (2017) 2:738–46. doi: 10.1016/S2468-1253(17)30154-1, PMID: 28690131

[177] Dekker PJ Koenders D Bruins MJ. Lactose-free dairy products: market developments, production, nutrition and health benefits. Nutrients. (2019) 11:551. doi: 10.3390/nu11030551, PMID: 30841534 PMC6471712

[178] Li A Zheng J Han X Yang S Cheng S Zhao J . Advances in low-lactose/lactose-free dairy products and their production. Foods. (2023) 12:2553. doi: 10.3390/foods12132553, PMID: 37444291 PMC10340681

[179] Dekker P. Enzymes exogenous to milk in dairy technology: β-d-galactosidase. (Amsterdam, Netherlands: Elsevier) (2022).

[180] Yamamoto E Watanabe R Ichimura T Ishida T Kimura K. Effect of lactose hydrolysis on the milk-fermenting properties of *Lactobacillus delbrueckii* ssp. bulgaricus 2038 and Streptococcus thermophilus 1131. J Dairy Sci. (2021) 104:1454–64. doi: 10.3168/jds.2020-19244, PMID: 33309355

[181] Marco ML Sanders ME Gänzle M Arrieta MC Cotter PD De Vuyst L . The international scientific Association for Probiotics and Prebiotics (ISAPP) consensus statement on fermented foods. Nat Rev Gastroenterol Hepatol. (2021) 18:196–208. doi: 10.1038/s41575-020-00390-5, PMID: 33398112 PMC7925329

[182] Aljutaily T Huarte E Martinez-Monteagudo S Gonzalez-Hernandez JL Rovai M Sergeev IN. Probiotic-enriched milk and dairy products increase gut microbiota diversity: a comparative study. Nutr Res. (2020) 82:25–33. doi: 10.1016/j.nutres.2020.06.017, PMID: 32949953

[183] van Deuren T Blaak EE Canfora EE. Butyrate to combat obesity and obesity-associated metabolic disorders: current status and future implications for therapeutic use. Obes Rev. (2022) 23:e13498. doi: 10.1111/obr.13498, PMID: 35856338 PMC9541926

[184] Kamel DG Hammam AR Alsaleem KA Osman DM. Addition of inulin to probiotic yogurt: viability of probiotic bacteria (*Bifidobacterium bifidum*) and sensory characteristics. Food Sci Nutr. (2021) 9:1743–9. doi: 10.1002/fsn3.2154, PMID: 33747485 PMC7958560

[185] Gibson GR Hutkins R Sanders ME Prescott SL Reimer RA Salminen SJ . Expert consensus document: the international scientific Association for Probiotics and Prebiotics (ISAPP) consensus statement on the definition and scope of prebiotics. Nat Rev Gastroenterol Hepatol. (2017) 14:491–502. doi: 10.1038/nrgastro.2017.75, PMID: 28611480

[186] Ho SW El-Nezami H Shah NP. The protective effects of enriched citrulline fermented milk with *Lactobacillus helveticus* on the intestinal epithelium integrity against *Escherichia coli* infection. Sci Rep. (2020) 10:499. doi: 10.1038/s41598-020-57478-w, PMID: 31949265 PMC6965087

[187] Putri I Haskito A Permana D. Effect of goat Milk yogurt fortified with red Rice bran flour on SGPT levels of rats (*Rattus norvegicus*) model diabetes mellitus induced Streptozotocin. J Phys Conf Ser. (2020) doi: 10.1088/1742-6596/1430/1/012008

[188] Mahdi C Ajeng Erika PH Ningtyas CK. Effect of giving goat Milk yogurt with the fortification of red Rice bran flour to kidney histopathology and creatinine level in the white rat (*Rattus Norvegicus*) model of type 1 diabetes mellitus Streptozotocin (STZ) induction. Int J Pharm Res. (2019) 11:09752366.

[189] Oliveira RPDS Florence ACR Perego P De Oliveira MN Converti A. Use of lactulose as prebiotic and its influence on the growth, acidification profile and viable counts of different probiotics in fermented skim milk. Int J Food Microbiol. (2011) 145:22–7. doi: 10.1016/j.ijfoodmicro.2010.11.011 21144608

[190] Gannasin SP Mustafa S Adzahan NM Muhammad K. In vitro prebiotic activities of tamarillo (*Solanum betaceum* Cav.) hydrocolloids. J Funct Foods. (2015) 19:10–9. doi: 10.1016/j.jff.2015.09.004

[191] Wongputtisin P Khanongnuch C. Prebiotic properties of crude oligosaccharide prepared from enzymatic hydrolysis of basil seed gum. Food Sci Biotechnol. (2015) 24:1767–73. doi: 10.1007/s10068-015-0230-9

[192] Hernandez LM. Mucilage from chia seeds (*Salvia hispanica*): Microestructure, physico-chemical characterization and applications in food industry. Chile: Pontificia Universidad Catolica de Chile (2012).

[193] Xing X Hsieh YS Yap K Ang ME Lahnstein J Tucker MR . Isolation and structural elucidation by 2D NMR of planteose, a major oligosaccharide in the mucilage of chia (*Salvia hispanica* L.) seeds. Carbohydr Polym. (2017) 175:231–40. doi: 10.1016/j.carbpol.2017.07.059, PMID: 28917861

[194] Hovjecki M Radovanovic M Levic SM Mirkovic M Peric I Miloradovic Z . Chia seed mucilage as a functional ingredient to improve quality of goat milk yoghurt: effects on rheology, texture, microstructure and sensory properties. Fermentation. (2024) 10:382. doi: 10.3390/fermentation10080382

[195] Du X Myracle AD. Fermentation alters the bioaccessible phenolic compounds and increases the alpha-glucosidase inhibitory effects of aronia juice in a dairy matrix following in vitro digestion. Food Funct. (2018) 9:2998–3007. doi: 10.1039/C8FO00250A, PMID: 29774337

[196] Gunes-Bayir A Bilgin MG Guclu D Pogda S Dadak A. Preparation and evaluation of novel functional fermented dairy products containing propolis and cinnamon. J Food Sci Technol. (2022) 59:2392–401. doi: 10.1007/s13197-021-05255-6, PMID: 34629508 PMC8487671

[197] de Almeida Brasiel PG Dutra Medeiros J Barbosa Ferreira Machado A Schuchter Ferreira M Gouveia Peluzio MC Potente Dutra Luquetti SC. Microbial community dynamics of fermented kefir beverages changes over time. Int J Dairy Technol. (2021) 74:324–31. doi: 10.1111/1471-0307.12759

[198] Albuquerque Pereira MF Matias Albuini F Gouveia Peluzio MC. Anti-inflammatory pathways of kefir in murine model: a systematic review. Nutr Rev. (2024) 82:210–27. doi: 10.1093/nutrit/nuad052, PMID: 37203423

[199] Suryani R Arfiansyah I Leonatra CY Febrisiantosa A, ed. Antioxidant activities of cell-free supernatant kefir obtained from two different kefir grains. AIP Conference Proceedings; (2024): AIP Publishing.

[200] Saleem K Ikram A Saeed F Afzaal M Ateeq H Hussain M . Nutritional and functional properties of kefir. Int J Food Prop. (2023) 26:3261–74. doi: 10.1080/10942912.2023.2280437

[201] Kim E-D Lee H-S Kim K-T Paik H-D. Antioxidant and angiotensin-converting enzyme (ACE) inhibitory activities of yogurt supplemented with Lactiplantibacillus plantarum NK181 and *Lactobacillus delbrueckii* KU200171 and sensory evaluation. Foods. (2021) 10:2324. doi: 10.3390/foods10102324, PMID: 34681373 PMC8534810

[202] Chang GR-L Cheng W-Y Fan H-C Chen H-L Lan Y-W Chen M-S . Kefir peptides attenuate atherosclerotic vascular calcification and osteoporosis in atherogenic diet-fed ApoE−/− knockout mice. Front Cell Dev Biol. (2023) 11:1158812. doi: 10.3389/fcell.2023.1158812, PMID: 37091976 PMC10117689

[203] Nurwantoro N Rizqiati H Karimy MF Wahyuningsih R Fauziah FR Ananda NSD ., eds. In vitro investigation of functional properties on cow milk and goat milk kefir whey with fermentation length treatment. AIP Conference Proceedings; (2024): AIP Publishing.

[204] Widodo W Fanani TH Fahreza MI Sukarno AS. Cholesterol assimilation of two probiotic strains of *Lactobacillus casei* used as dairy starter cultures. Appl Food Biotechnol. (2021) 8:103–12. doi: 10.22037/afb.v8i2.30661

[205] Mgbechidinma CL Adegoke CO Ogunbanwo ST. Lactic acid bacteria as bioactive potential against selected resistance Candida species and pathogenic bacteria. Int J Pharm Biol Sci Arch. (2020) 8:19–32. doi: 10.32553/ijpba.v8i2.165

[206] Moure MC Pérez Torrado R Garmendia G Vero S Querol A Alconada T . Characterization of kefir yeasts with antifungal capacity against *aspergillus* species. Int Microbiol. (2023) 26:361–70. doi: 10.1007/s10123-022-00296-z 36370206

[207] Saidi V Sheikh-Zeinoddin M Kobarfard F Soleimanian-Zad S. Bioactive characteristics of a semi-hard non-starter culture cheese made from raw or pasteurized sheep's milk. 3 Biotech. (2020) 10:1–8. doi: 10.1007/s13205-020-2075-z 32089980 PMC7000560

[208] Yang Y Xia Y Li C Wang G Xiong Z Song X . Metabolites, flavor profiles and ripening characteristics of Monascus-ripened cheese enhanced by Ligilactobacillus salivarius AR809 as adjunct culture. Food Chem. (2024) 436:137759. doi: 10.1016/j.foodchem.2023.137759, PMID: 37857204

[209] Sulejmani E Hayaloglu AA. Influence of starter culture on nitrogen fraction and volatile compounds in beaten cow's milk cheese. J Food Proc Preser. (2020) 44:e14689. doi: 10.1111/jfpp.14689

[210] Sun M Yu J Song Y Li X Mu G Tuo Y. Metabolomic analysis of fermented milk with *Lactobacillus delbrueckii* subsp. bulgaricus, Lacticaseibacillus paracasei cocultured with Kluyveromyces marxianus during storage. Food Bioscience. (2023) 54:1–9. doi: 10.1016/j.fbio.2023.102901

[211] Peng J Ma L Kwok LY Zhang W Sun T. Untargeted metabolic footprinting reveals key differences between fermented brown milk and fermented milk metabolomes. J Dairy Sci. (2022) 105:2771–90. doi: 10.3168/jds.2021-20844, PMID: 35094863

[212] Huang P Yu L Tian F Zhao J Zhang H Chen W . Untargeted metabolomics revealed the key metabolites in milk fermented with starter cultures containing *Lactobacillus plantarum* CCFM8610. Lwt. (2022) 165:1–9. doi: 10.1016/j.lwt.2022.113768

[213] Li D Peng J Kwok L-y Zhang W Sun T. Metabolomic analysis of *Streptococcus thermophilus* S10-fermented milk. Lwt. (2022) 161:1–10. doi: 10.1016/j.lwt.2022.113368

[214] Zha M Li K Zhang W Sun Z Kwok L-Y Menghe B . Untargeted mass spectrometry-based metabolomics approach unveils molecular changes in milk fermented by *Lactobacillus plantarum* P9. Lwt. (2021) 140:1–9. doi: 10.1016/j.lwt.2020.110759

[215] Xia Y Yu J Miao W Shuang Q. A UPLC-Q-TOF-MS-based metabolomics approach for the evaluation of fermented mare's milk to koumiss. Food Chem. (2020) 320:126619. doi: 10.1016/j.foodchem.2020.126619, PMID: 32203836

[216] Hou Q Li C Liu Y Li W Chen Y Siqinbateer . Koumiss consumption modulates gut microbiota, increases plasma high density cholesterol, decreases immunoglobulin G and albumin. J Funct Foods. (2019) 52:469–78. doi: 10.1016/j.jff.2018.11.023

