# Supplementary material for: Certain fermented dairy foods as a source of multibiotics and multimetabolites: a comprehensive review
Source: Front Nutr. 2025 Nov 6;12:1678150. doi: 10.3389/fnut.2025.1678150 (PMC12633755; doi:10.3389/fnut.2025.1678150)
Supplement: Supplementary file 1 [file Table_1.docx]

**Supplementary Table 1.** Application of metabolomics in fermented dairy products

| **Food** | **Method** | **Condition** | **Microorganisms involved** | **Metabolites** | **Pathways** | **Proposed health effects** | **Reference** |
| --- | --- | --- | --- | --- | --- | --- | --- |
| Fermented milk | LC-MS, UPLC | Effect of co-culture on storage (after 30 days) | *Lacticaseibacillus paracasei* PC-01 | Hypoxanthine, Thiamine, 5-Aminopentanoic acid, O-Acetylleucine, Hydrogen phosphate, N-Acetyl-L-aspartic acid, N-acetylmannosamine, Arbutin, Beta-Carboline, D-Tagotose, D-2,3,Dihydroxypropanoic acid, 4-Hydroxyproline, Acetamimophen, Norvaline, Xanthine, Oleic acid etc. | Biosynthesis of nucleotide  sugars, D-amino acid metabolism, arginine and proline metabolism, TCA cycle, methane metabolism,  thiamine metabolism,  nucleotide metabolism, and carbon metabolism | Not determined | (34) |
|  |  |  | *Lacticaseibacillus paracasei* PC-01, *Bifidobacterium adolescentis* B8589 | Xantine, GABA, Methylglutaric acid, L-Malic acid, Dehydroascorbic acid, Hypoxanthine, N-acetiyl-L-methionine, Leucyl-phenylalanine, Thiamine, Alpha-Linoleic acid | Purine metabolism,  cysteine and methionine metabolism, alanine, aspartate and glutamate metabolism, glyoxylate and dicarboxylate metabolism, ABC transporters, TCA cycle, methane metabolism, nucleotide metabolism,  thiamine metabolism,  sphingolipid metabolism, glycerophospholipid metabolism, metabolic pathways, biosynthesis of secondary metabolites, biosynthesis of cofactors, and carbon metabolism | Because of GABA content, it has promising effects on depression, insomnia and diabetes |  |
| Fermented milk | UHPLC-Q-TOF-MS | Effect of co-culture on storage (after 13 days) | 2J group: *Lactobacillus delbrueckii* subsp. *bulgaricus* DPUL-F36, *Lacticaseibacillus paracasei* DPUL-F115) | Higher in 3J than 2J:  Amino acids and metabolites, benzene and substituted derivatives, aldehyde, ketones, esters, and organic acid and  its derivatives etc. |  | Not determined | (199) |
|  |  |  | 3J group:  *Lactobacillus delbrueckii* subsp. *bulgaricus* DPUL-F36  and *Lacticaseibacillus paracasei* DPUL-F115) and *Kluyveromyces marxianus* 39 |  | Phenylalanine, tyrosine, and tryptophan, biosynthesis, glycolysis/gluconeogenesis, galactose metabolism, biosynthesis of cofactors etc. | Hydrolysis of lactose |  |
| Fermented milk | UPLC-QE-MS | Effect of storage time (0, 36, 60 and 72 hours) | *Lacticaseibacillus paracasei* PC-01 and  *Bifidobacterium adolescentis* B8589 | Differences between time points:  Lipids and lipid-like molecules (e.g. capric acid), organic acids and derivatives (e.g. pyruvate, GABA), organic oxygen compounds,  and organ heterocyclic compounds, followed by phenylpropanoids  and polyketides, benzenoids, alkaloids and derivatives etc. | Biosynthesis of unsaturated fatty acids  Primary bile acid biosynthesis  Glycerophospholipid metabolism  Amino sugar and nucleotide sugar metabolism  Purine metabolism Pentose phosphate pathway  Sphingolipid metabolism  Phosphatidylinositol signalling system  Inositol phosphate metabolism etc. | Pyruvate, GABA and capric acid related health effects | (9) |
| Fermented brown milk and fermented milk | UPLC-Q-TOF MS | Comparison of fermented brown and fermented milk | *Lactobacillus bulgaricus* ND02 | Differences between fermented brown and fermented milk:  l-Lysine, (R)-4'-  Phosphopantothenoyl-lcysteine, Carboxymethyl lysine, l-Methionine S-oxide, Glutaurine, l-Glutamyl-l-cysteinylglycin, Methylglyoxal, Glyoxal, 2,3-Pentanedione, Glycerophosphocholine, N6-Acetyl-LL-2,6-  Diaminopimelate, N-Acetylmuramic acid 6-phosphate, 3-Hydroxybutanoic acid, Butyryl-CoA, Cinnabarinic acid, Spermidine | Differences between fermented brown and fermented milk:  Glycoliysis/Gluconeogenesis, Purine metabolism, Pyruvate metabolism, Phenylalanine metabolism Valine, leucine and isoleucine degradation, Riboflavin metabolism, | Increase in carboxymethyllysine  might have negative health effect.  Differential abundant  metabolites might have different nutritional qualities | (200) |
| Fermented milk | LC-MS/MS | Effect of *L. plantarum* CCFM8610 on fermentation | S batch: *Streptococcus thermophiles*  SL batch: *Streptococcus thermophilus* and *Lactiplantibacillus plantarum* | Differences between S vs SL batch:  Benzoic acid, D-tryptophan, 7-methylguanine, D-phenyllactic acid, hippuric acid, creatinine, choline, 6-hydroxycaproic acid, N-oleoylethanolamine, and 1-methyladenine | Phenylalanine metabolism; serine, glycine, threonine metabolism, glycerophospholipid metabolism etc. | Physiological  functions regulating gut microorganisms and alleviating disease | (201) |
| Fermented milk | UPLC-QTOF MS | Effect of fermentation | *Streptococcus thermophilus* S10 | Differences between milk and fermented milk:  Peptides, tyrosin, cysteine, L-serine, aspartate, Purine, Ester substrates, succinate, riboflavin, alpha-eleostearic acid, myristic acid, lauric acid, oleic acid, palmitic acid | Fatty acid metabolism  pathway, fatty acid biosynthesis pathway, tyrosine metabolism pathway, glycine and serine metabolism  pathway, glutamate metabolism pathway, and homocysteine degradation  pathway, riboflavin metabolism pathway | Small  peptides may have beneficial functions | (202) |
| Fermented milk | UPLC-Q-TOF-MS/MS | Effect of fermentation | *Lactiplantibacillus plantarum* P9 | Fatty acid and related substances (oleic, stearic, myristic, malic acid etc.), Peptides, leucine, lactose, pantothenic acid, aldehyde, ketone and related substances | Phenylalanine metabolism | Not determined | (203) |
| Fermented mare milk (Koumiss) | UPLC-Q-TOF-MS/MS | Effect of fermentation | *Lactobacillus delbrueckii*  subsp. *bulgaricus*,  *Lactobacillus casei*,  *Bifidobacterium lactis*, *Streptococcus thermophiles*,  *Kluyveromyces lactis* CICC  1772 | Glycerophospholipids, fatty acyls, carboxylic acids and derivatives, benzenoids, glycosides, organoheterocyclic compounds, glycerolipids, alcohols, lactones, carbonyl compounds p-Pyruvate, 20-hydroxy arachidonic  acid (20-hete), 4-aminobutanoate, uracil, acetoacetate | Fatty acid biosynthesis, b-alanine metabolism pathway,  pyrimidine  metabolism pathway, Aminoacyl-tRNA  biosynthesis, c-5-branched dibasic acid  metabolism pathway, arginine,  proline metabolism pathway, valine,  leucine and isoleucine biosynthesis,  vascular smooth muscle contraction etc. | Not determined | (204) |
| Koumiss | UPLC/ESI-QTOF/MS | Effect of fermentation | *Lactobacillus helveticus*, *Lactobacillus kefiranofaciens*,  *Lactobacillus kefiri*, *Streptococcus parauberis* and  *Lactococcus lactis* | Methionyl-asparagine, 2′-Deoxy-5′-adenylic acid, S-Adenosyl-L-methionine, Carnosine, Lysophosphatidylinositol, Prolyl-asparagine, Cytosine, Lysylvaline, Cholic acid, Histidylhistidine, Pyroglutamic acid, Glutamic acid, 6-Acetamidohexanoic acid, Glycyl-N∼5∼  (diaminomethylene) ornithine, Methionyl-phenylalanine, D-Tyrosyl-L-allo isoleucine-Lleucine, Pyridoxine, Hydroxyphenylacetaldehyde, Hexanoic acid, Biotin, S-Adenosylmethioninamine, Phenylalanyl-asparagine | - | Increases plasma high density cholesterol, decreases immunoglobulin G and albumin by stimulation of gut microbiota | (205) |
| Yogurt | GC-MS | *Effect of different type of milk (cow milk vs goat milk) on the metabolite profile in yogurt during storage (14 days)* | *Lactobacillus delbrueckii*  *subsp. bulgaricus* and *S. thermophilus* | The increased levels of free amino acids and dipeptides, carboxylic acids and fatty acid derivatives in GY, whereas the elevated tri-peptide levels in CY | glycine, serine and  threonine metabolism, amino-acyl tRNA biosynthesis, pentose  phosphate pathway, cysteine and methionine metabolism and  glycrophospholipid metabolism | - | (59) |
| Yogurt | GC-MS | Effect of different type of milk (sheep milk vs goat milk) on the metabolite profile in yogurt , one day  post-manufacture | *Streptococcus thermophilu*s and Lactobacillus  delbrueckii subsp. *bulgaricus* | Goat yogurt has higher levels of free amino acids, such as isoleucine, valine, phenylalanine, threonine, serine, methionine, lysine,  glutamine/glutamic acid, proline, aspartic acid, together with GABA  and pyroglutamic acid, uracil, bphenyllactic  acid, a-hydroxyisocaproic acid, and creatinine, while  sheep yogurt samples were richer in N-acetylglucosamine, N-acetylgalactosamine,  a-hydroxyisobutyric acid, myo-inositol, xylose, ribonic acid,  and glucitol | - | Pyroglutamic acid has an antimicrobial  activity and anti-diabetic effect, b-Phenyllactic acid has antimicrobial properties, GABA can participate to several metabolic pathways in  humans promoting health by its anti-stress, anti-hypertensive, and  anti-diabetic properties | (60) |
| Yogurt | NMR | Monitor the changes in two differently heat-treated milks (99°C or 105°C) during fermentation from 0 to 24 hours using three different starter cultures | *L. delbrueckii* ssp. *bulgaricus,*  *S. thermophilus* and their combination | The symbiotic relationship between Lactobacillus and Streptococcus is driven by their complementary metabolic activities. While Lactobacillus requires formate for growth but cannot produce it, Streptococcus generates formate from pyruvate, thereby supporting Lactobacillus proliferation. In return, Lactobacillus contributes to product quality by hydrolyzing milk proteins into amino acids, enhancing the nutritional value of the final product | - | - | (61) |
| Yogurt | LC-MS and NMR | chemical composition of chemically acidified yoghurt-like whole milk without bacterial  fermentation, and a fermented and then high-temperature pasteurized  (95 ◦C, 30 sec) whole milk yoghurt, fresh commercial whole milk yoghurt and whole milk | *S. thermophilus* and *L. delbrueckii* subsp. *bulgaricus* | Significantly higher levels of peptides in fermented yogurt compared to chemically acidified milk and whole milk. Chemically acidified and heat-inactivated fermented yogurts had the highest number of unique peptides, indicating distinct peptide profiles across products. Peptides from chemically acidified milk and whole milk were mainly composed of lysine, arginine, and leucine. Heat-inactivated fermented yogurt showed higher concentrations of amino acids such as valine, methionine, leucine, glutamate, and alanine compared to fermented yogurt | Peptides and sugar metabolism | Antihypertensive, antimicrobial, antioxidant and Immunomodulatory bioactive peptides | (55) |
| Yogurt | LC-MS / GC-MS | The metabolomic profiles in  yogurts induced by multistrain probiotics vs conventional yogurt under different fermentation temperatures  (37°C and 42°C) | *Probiotics: Lactobacillus*  *casei* Zhang (LCZ) and *Bifidobacterium lactis* V9  (V9)  Conventional starter cultures: *Lactobacillus delbrueckii* ssp. *Bulgaricus, S. thermophilus* | Pathways such as valine (Val), leucine (Leu), isoleucine (Ile) degradation, pyruvate metabolism, folate biosynthesis, and glycerolipid metabolism were enriched in probiotic yogurt compared to conventional yogurt, Pathways like **phosphonate and phosphinate metabolism, polyketide sugar unit biosynthesis, acarbose biosynthesis, riboflavin metabolism,** and **methane metabolism** were enriched in probiotic yogurt compared to conventional yogurt, The multistrain probiotic yogurt (MSC) displayed more complex and diverse metabolic profiles, especially at 42°C, indicating that fermentation temperature plays a significant role in influencing the metabolic composition | AA biosynthesis pathways; alanine,  aspartate and glutamate metabolism; tyrosine metabolism;  valine, leucine, and isoleucine degradation  , pentose phosphate pathway | - | (64) |
| Yogurt | LC-MS | The effects of  adding LS to milk at concentrations (0 %, 2 %, 5 % and 8 % w/v) for yogurt production, in yogurt microbial composition and metabolomics | *Lactobacillus delbrueckii* ssp. *bulgaricus and*  *Streptococcus thermophilus* | Variation of metabolites, mainly included organic acids and their derivatives,  lipids and lipid-like molecules, organic heterocyclic compounds, phenylpropanoids  and polyketides, organic oxygen compounds, and benzene ring-type compounds, in yogurt with different amounts of  added LS.  Several amino acids and their derivatives were significantly up regulated in the metabolites of  yogurt added with different concentrations of LS.  Several sphingolipids were also upregulated.  The phenolic compound  3,4-  dihydroxybenzylamine showed a very significant up-regulation  In addition, a significant down regulation  of phenylpropanoids was also observed. | Phenylalanine, tyrosine and tryptophan biosynthesis, arginine and proline metabolism, D-glutamine and D-glutamate metabolism, pyrimidine metabolism, glutathione metabolism, thiamine metabolism, nicotinate and nicotinamide metabolism | Amino acids can influence  the flavor of fermented products, regulate intestinal health,  improve blood flow, boost immunity, and improve brain cell metabolism.  Sphingolipids are  the major lipids of biological membranes and are involved in physiological  functions such as cell signaling, cell proliferation, and differentiation.  3,4-  dihydroxybenzylamine, which could significantly improve the therapeutic  effect on melanoma | (68) |
| Sourcream | UPLC-Q-TOF MS^E^ | Identifying metabolite differences between sourcream and butter | 48 isolates by pure culture: *Enterococcus*cass liflavus (2 strains), *Enterococcus* italicus (2 strains), *Enterococcus* faecium (2 strains), Lactobacillus fermentum (2 strains), Lactobacillus paracasei (1 strains), Lactobacillus plantarum (7 strains), Lactobacillus curvatus (1 strains), Lactococcus lactis (16 strains), Lactococcus garvieae (2 strains), Leuconostoc mesenteroides (3 strains), Leuconostoc garlicum (2 strains), Leuconostoc lactis (1 strains), Pediococcus acidilactici (2 strains), Streptococcus thermophilus (4 strains) ve Weissella hellenica (1 strains) | Tyr-cys, Uridine, His-asn, Lys-val, Decanoic acid, Met-tyr, Isohexanal, Lys-lys, Palmitic acid, 2’-Deoxycytidine, L-Xylulose, Leu-val, Serine, L-Cysteine, Cystine, Aspartic acid, L-Lysine, L-Alanine, gly-ser-pro-met-phe-ala-val | - | - | (76) |
| Fermented milk | LC-MS, UPLC | Effect of co-culture on storage (after 30 days) | *Lacticaseibacillus paracasei* PC-01 | Hypoxanthine, Thiamine, 5-Aminopentanoic acid, O-Acetylleucine, Hydrogen phosphate, N-Acetyl-L-aspartic acid, N-acetylmannosamine, Arbutin, Beta-Carboline, D-Tagotose, D-2,3,Dihydroxypropanoic acid, 4-Hydroxyproline, Acetamimophen, Norvaline, Xanthine, Oleic acid etc. | Arginine and proline metabolism, biosynthesis of nucleotide  sugars, microbial metabolism in diverse environments, and D-amino acid  metabolism  TCA cycle, methane metabolism,  thiamine metabolism, microbial metabolism in diverse environments,  nucleotide metabolism, and carbon metabolism, | - | (34) |
|  |  |  | *Lacticaseibacillus paracasei* PC-01 and *Bifidobacterium adolescentis* B8589 | Xantine, GABA, Methylglutaric acid, L-Malic acid, Dehydroascorbic acid, Hypoxanthine, N-acetiyl-L-methionine, Leucyl-phenylalanine, Thiamine, Alpha-Linoleic acid | Purine metabolism,  thiamine metabolism,  cysteine and methionine metabolism, alanine, aspartate and  glutamate metabolism, glyoxylate and dicarboxylate metabolism,  sphingolipid metabolism, glycerophospholipid metabolism, ABC transporters,  metabolic pathways, biosynthesis of secondary metabolites,  biosynthesis of cofactors, TCA cycle, methane metabolism,  thiamine metabolism, microbial metabolism in diverse environments,  nucleotide metabolism, and carbon metabolism | Because of GABA content, promising effects on depression, insomnia and diabetes |  |
| Fermented milk | UHPLC-Q-TOF-MS | Effect of co-culture on storage (after 13 days) | 2J group: *Lactobacillus delbrueckii* subsp. *bulgaricus* DPUL-F36  and *Lacticaseibacillus paracasei* DPUL-F115) | Higher in 3J than 2J:  Amino acids and metabolites, benzene and substituted derivatives, aldehyde, ketones, esters, and organic acid and  its derivatives Lysylglutamic  acid (MW0108094), Leu-Leu-Leu-Pro-Gly (MW0152400), Phe-  Leu-Leu (MW0155252), H-Leu-val-OH (MW0107766), His-Gly-Val-Asp-  Lys (MW0150950), glutamine, citric acid etc. |  | - | (199) |
|  |  |  | 3J group:  *Lactobacillus delbrueckii* subsp. *bulgaricus* DPUL-F36  and *Lacticaseibacillus paracasei* DPUL-F115) and *Kluyveromyces marxianus* 39 |  | Glycolysis/Gluconeogenesis,  Galactose metabolism, Phenylalanine, tyrosine, and tryptophan biosynthesis, and Biosynthesis of cofactors etc. | Hydrolysis of lactose |  |
| Fermented milk | UPLC-QE-MS | Effect of storage time (0, 36, 60 and 72 hours) | *Lacticaseibacillus paracasei* PC-01 and  *Bifidobacterium adolescentis* B8589 | Differences between time points:  Lipids and lipid-like  Molecules (e. g. capric acid), organic acids and derivatives (e.g. Pyruvate, GABA), organic oxygen compounds,  and organ heterocyclic compounds, followed by phenylpropanoids  and polyketides, benzenoids, alkaloids and derivatives etc. | Biosynthesis of unsaturated fatty acids  Primary bile acid biosynthesis  Sphingolipid metabolism  Glycerophospholipid metabolism  Pentose phosphate pathway  Sphingolipid metabolism  Phosphatidylinositol signaling system  Inositol phosphate metabolism  Amino sugar and nucleotide sugar metabolism  Purine metabolism etc. | Pyruvate, GABA and capric acid related health effects | (9) |
| Fermented brown milk and fermented milk | UPLC-Q-TOF MS | Comparison of fermented brown and fermented milk | *Lactobacillus bulgaricus* ND02 | Differences between fermented brown and fermented milk:  l-Lysine, (R)-4'-  Phosphopantothenoyl-lcysteine, Carboxymethyl lysine, l-Methionine S-oxide, Glutaurine, l-Glutamyl-l-cysteinylglycin, Methylglyoxal, Glyoxal, 2,3-Pentanedione, Glycerophosphocholine, N6-Acetyl-LL-2,6-  Diaminopimelate, N-Acetylmuramic acid 6-phosphate, 3-Hydroxybutanoic acid, Butyryl-CoA, Cinnabarinic acid, Spermidine | Differences between fermented brown and fermented milk:  Valine, leucine and isoleucine degradation, Riboflavin metabolism, Glycoliysis/Gluconeogenesis, Purine metabolism, Pyruvate metabolism, Phenylalanine metabolism | Increase in carboxymethyllysine  might have negative health effect.  Differential abundant  metabolites might have different nutritional qualities | (200) |
| Fermented milk | LC-MS/MS | Effect of L. plantarum CCFM8610 on fermentation | S batch: *Streptococcus thermophiles*  SL batch: *Streptococcus thermophilus* and *Lactiplantibacillus plantarum* | Differences between S vs SL batch:  Benzoic acid, D-phenyllactic acid,  hippuric acid, creatinine, D-tryptophan, 7-methylguanine, choline, 6-  hydroxycaproic acid, N-oleoylethanolamine, and 1-methyladenine.  Among these differential metabolites, benzoic acid, 6-hydroxycaproic  acid, D-phenyllactic acid, hippuric acid | Phenylalanine metabolism; glycine, serine, and threonine metabolism,  glycerophospholipid metabolism etc. | Physiological  functions regulating gut microorganisms and alleviating disease | (201) |
| Fermented milk | UPLC-QTOF MS | Effect of fermentation | *Streptococcus thermophilus* S10 | Differences between milk and fermented milk:  Peptides, Purine, Ester substrates, succinate, riboflavin, oleic acid, palmitic acid, alpha-eleostearic acid, myristic acid, lauric acid, tyrosin, cysteine, L-serine, aspartate | Fatty acid metabolism  pathway, fatty acid biosynthesis pathway, riboflavin metabolism  pathway, tyrosine metabolism pathway, glycine and serine metabolism  pathway, glutamate metabolism pathway, and homocysteine degradation  pathway | Small  peptides detected in fermented milk may have beneficial functions | (202) |
| Fermented milk | UPLC-Q-TOF-MS/MS | Effect of fermentation | *Lactiplantibacillus plantarum* P9 | Fatty acid and related substances (oleic, stearic, myristic, malic acid etc.), Peptides, leucine, lactose, pantothenic acid, aldehyde, ketone and related substances | Phenylalanine metabolism | - | (203) |
| Fermented mare milk (Koumiss) | UPLC-Q-TOF-MS/MS | Effect of fermentation | *Lactobacillus delbrueckii*  subsp*. bulgaricus,*  *Streptococcus thermophiles,*  *Lactobacillus casei,*  *Bifidobacterium lactis,*  *Kluyveromyces lactis* CICC  1772 | Glycerophospholipids, fatty acyls,  carboxylic acids and derivatives,  benzenoids, glycosides,  organoheterocyclic compounds,  glycerolipids, alcohols, lactones,  carbonyl compounds p-Pyruvate, 20-hydroxy arachidonic  acid (20-hete), 4-aminobutanoate,  uracil, acetoacetate, 4-aminobutanoate,  acetoacetate, uracil | b-Alanine metabolism pathway, fatty  acid biosynthesis, pyrimidine  metabolism pathway, Aminoacyl-tRNA  biosynthesis, c-5-branched dibasic acid  metabolism pathway, arginine,  proline metabolism pathway, valine,  leucine and isoleucine biosynthesis,  vascular smooth muscle contraction etc. | - | (204) |

| Koumiss | UPLC/ESI-QTOF/MS | Effect of fermentation | *Lactobacillus helveticus, Lactobacillus kefiranofaciens,*  *Lactobacillus kefiri*, *Streptococcus parauberis* and  *Lactococcus lactis* | Methionyl-asparagine, 2′-Deoxy-5′-adenylic acid, S-Adenosyl-L-methionine, Carnosine, Lysophosphatidylinositol, Prolyl-asparagine, Cytosine, Lysylvaline, Cholic acid, Histidylhistidine, Pyroglutamic acid, Glutamic acid, 6-Acetamidohexanoic acid, Glycyl-N∼5∼  (diaminomethylene) ornithine, Methionyl-phenylalanine, D-Tyrosyl-L-allo isoleucine-Lleucine, Pyridoxine, Hydroxyphenylacetaldehyde, Hexanoic acid, Biotin, S-Adenosylmethioninamine, Phenylalanyl-asparagine | - | Increases plasma high density cholesterol, decreases immunoglobulin G and albumin by stimulation of gut microbiota | (205) |
| --- | --- | --- | --- | --- | --- | --- | --- |
